# Recycling of collagen from solid tannery waste and prospective utilization as adhesives.

**DOI:** 10.12688/f1000research.155450.1

**Published:** 2024-10-14

**Authors:** Nelly Esther Flores Tapia, Hannibal Brito Moina, Rodny Peñafiel, Lander Vinicio Pérez Aldás

**Affiliations:** 1 Research and Development Directorate, Technical University of Ambato, Ambato, Tungurahua, Ecuador; 2 Facultad de Ciencias, Escuela Superior Politecnica de Chimborazo, Riobamba, Chimborazo Province, Chimborazo, Ecuador; 3 Food and Biotechnology, Technical University of Ambato, Ambato, Tungurahua, Ecuador

**Keywords:** collagen; solid tannery wastes; animal glue; collagen adhesives; bio-adhesive, recycled

## Abstract

This study explores the innovative potential of recycled collagen derived from tannery waste for use in high-performance adhesive formulations. The leather industry generates significant amounts of solid waste, primarily from chromium-tanned leather, which poses substantial environmental challenges. Recent advancements in recycling techniques have opened new avenues for repurposing this waste, particularly through collagen extraction, which comprises about 30-35% of tannery residues. This research systematically reviews the methods and applications of collagen extraction, highlighting the material’s versatility and environmental benefits when used as a bio-adhesive.

The review identifies key challenges such as low water resistance, shear strength, and adhesiveness in collagen-based adhesives compared to synthetic counterparts. However, innovative solutions are emerging, including the incorporation of silane coupling agents and cross-linking technologies that significantly improve the water resistance and mechanical properties of these adhesives. Economic analyses further support the viability of using tannery waste-derived collagen in adhesive production, aligning with global sustainability goals and reducing reliance on petrochemical-based adhesives.

Despite these advancements, the transition from laboratory research to commercial applications remains a significant challenge. Current studies primarily focus on small-scale experiments, with limited pilot-scale studies available. Nonetheless, the potential for collagen-based adhesives to replace harmful chemicals in industrial applications is promising, especially in sectors requiring biodegradable and non-toxic materials. This review concludes that while significant progress has been made, further research is necessary to overcome existing limitations and fully realize the commercial potential of collagen-based adhesives derived from tannery waste.

## Introduction

**Figure 1.**
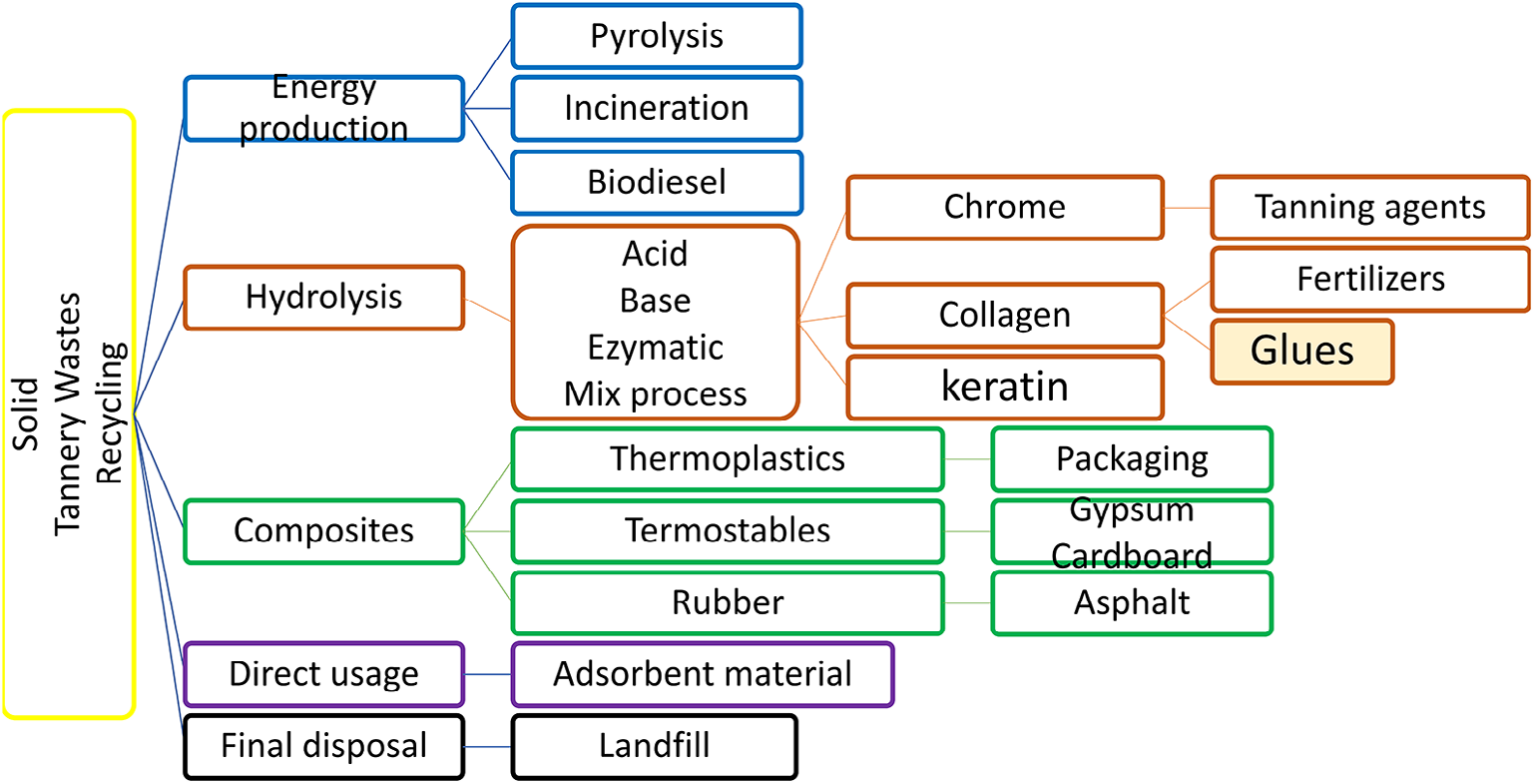
Brief description of processes applied to recycle solid tannery wastes. ^4^^–^^22^

The leather industry, particularly chromium-based tanning, generates substantial solid waste, including chromium sludge, chrome-tanned leather shavings, and trimmings, with only 20% of raw material converted into leather. ^1^^–^^4^ This results in significant waste, rich in collagen, which is often discarded in landfills due to the absence of cost-effective recycling programs. ^5^^–^^7^ Solid tannery waste, comprising around 25% untreated skin, contains approximately 30% to 35% collagen and 1.5% chromium, underscoring its potential for resource recovery. ^8^ India alone produces 0.02 million tons of chromium shavings annually (0.8 million tons of chromed leather trimmings per year), indicating a large potential resource for recycling into valuable products like renewed leather, ^9^ fertilizers in agriculture, composting, ^10^ formulation of composite materials, ^11^^,^^12^ production of biodiesel, ^13^^–^^17^ and extraction of raw materials such as keratin, ^18^ chromium, ^19^ and collagen ^20^^,^^21^ (see Figure 1).

However, synthetic adhesives have largely replaced animal glues due to concerns over cost, availability, and consistency. Despite this, synthetic adhesives are highly polluting, non-biodegradable, and dependent on petroleum, underscoring the need for eco-friendly alternatives. Collagen-modified adhesives, especially those derived from tannery waste, present a promising solution by offering both environmental benefits and effective adhesive properties.

Historically, collagen sourced from animal tissues like cartilage and tendons has long been used as an adhesive due to its environmentally friendly and non-toxic properties. ^22^ Over time, animal collagen has been applied in various forms, from craftsmanship to industrial processes, for its natural adhesive qualities. Its composition facilitates efficient and reversible adhesion in applications such as paper and cardboard, with low cure temperatures compared to dispersions or hot melts, setting it apart from synthetic counterparts. ^23^^,^^24^ Despite these advantages, synthetic chemicals supplanted animal glues at the beginning of the 20th century ^25^ due to drawbacks like cost, availability, animal welfare concerns, and inconsistencies in raw material composition that affect adhesive performance. ^26^ While synthetic adhesives provide several benefits, they are highly polluting, non-biodegradable, and reliant on petroleum, driving the search for eco-friendly alternatives. ^27^^–^^29^ Utilizing collagen extracted from tannery waste offers a promising avenue for producing effective adhesives while minimizing waste. ^30^

Although animal glues derived from tannery wastes have been explored as renewable alternatives, their viability is limited by the contamination present in tannery waste, rendering them unsuitable for applications like human tissue glues. ^31^^–^^33^ This review evaluates the potential of producing adhesives from collagen extracted from tannery wastes. It explores the methods, applications, and advancements in this area, with a particular focus on extraction techniques, adhesive formulation, and the associated environmental and economic benefits. By addressing gaps in current research, this review provides a comprehensive overview of the challenges and opportunities in utilizing tannery waste for sustainable adhesive production.

## Methods

Several online databases were searched to ensure comprehensive topic coverage: PubMed/MEDLINE, MDPI, Web of Science, Scopus, and ScienceDirect. The central theme was sourcing articles discussing adhesives and glues derived from solid tannery waste, focusing on innovations that utilize recycled collagen.

### Initial Broad Search on Scopus

- Terms Used: ‘collagen’ AND ‘adhesives’ AND NOT ‘dental’ AND NOT ‘human’ AND NOT ‘surgical’ AND NOT ‘skin’ AND NOT ‘healing.’

Outcomes: A total of 426 records were identified, excluding those discussing collagen adhesives for medical applications.

### Specific Search on Scopus

- Terms Used: ‘tannery’ AND ‘wastes’ AND (‘glues’ OR ‘adhesives’ OR ‘collagen’).

Outcome: 99 records identified.

### Focused Search on Web of Science

- Terms Used: ‘tannery’ AND ‘wastes’ AND ‘collagen-derived.’

Outcome: 93 articles were found.

Although numerous documents were retrieved, a limited number genuinely addressed the desired information. After removing the duplicates, 125 pertinent records were selected for the initial search.

***Timeline analysis***

**Figure 2.**
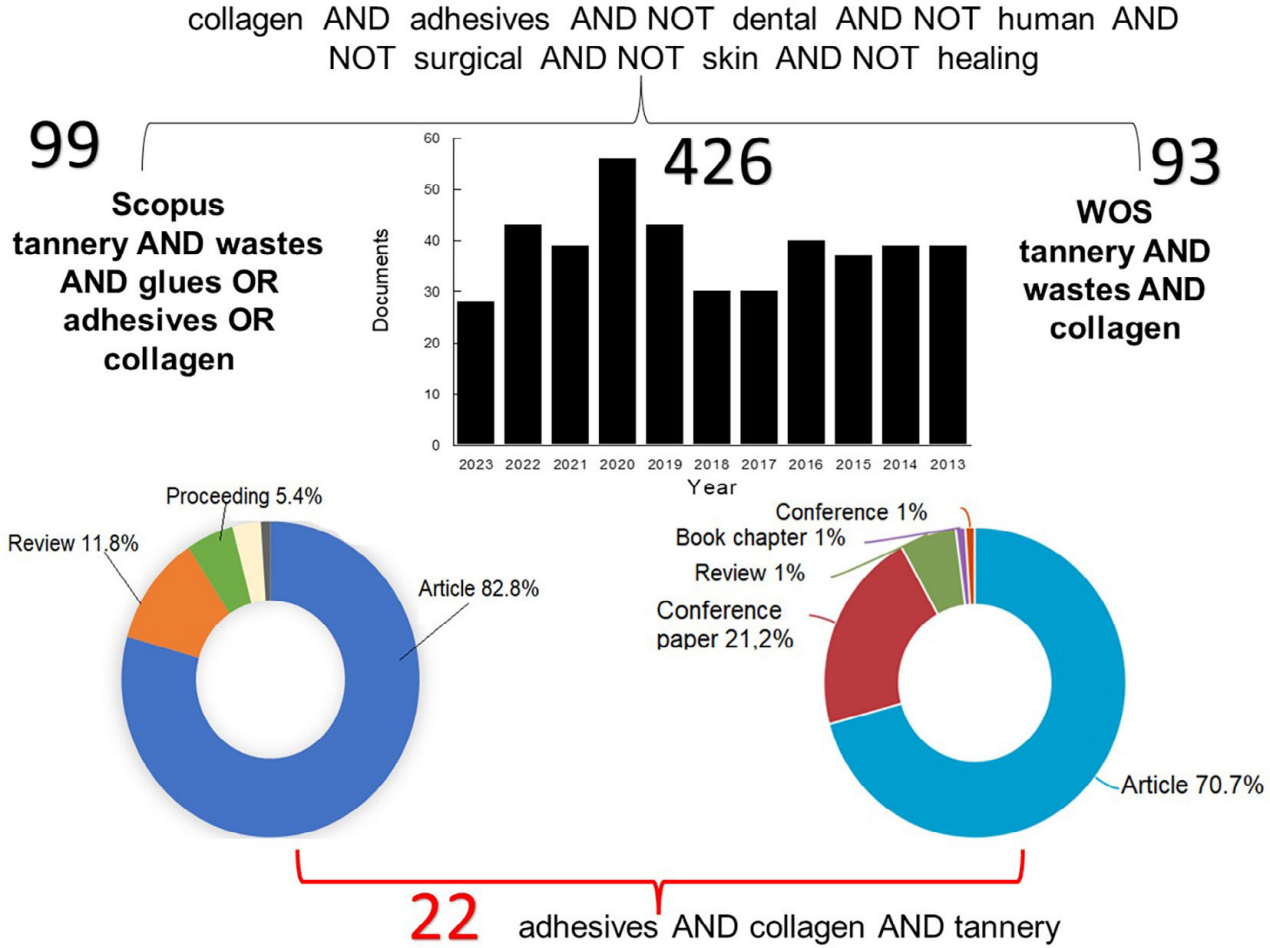
Summary of Research on Collagen-Based Adhesives from Tannery Waste in the Last Decade.

The search was confined to the past decade for in-depth scrutiny and to remain current. Of the shortlisted articles, 22 were subjected to qualitative analysis of the innovations in adhesive creation ( Figure 2). The data search was conducted from June 1 to August 24, 2023.

***Language***

Only documents in English.

### Additional Data Search

Economic, historical, and industrial data were extracted individually from specialized websites to provide a holistic perspective, focusing on the past 20 years.

The systematic search across multiple databases resulted in the identification of 125 pertinent records, which were further refined through qualitative analysis. These findings lay the foundation for evaluating the current state of research on recycled collagen-based adhesives, highlighting both the advancements and the ongoing challenges in this innovative field.

## Discussion

### Background on tannery waste and its environmental implications

Global trade in animal leather accounted for only 0.091% of the total international market in 2021, reaching a substantial value of $242.85 billion in 2022. ^34^ Notable exporters in this trade were Italy ($3.55 billion), the United States ($1.88 billion), Brazil ($1.45 billion), China ($1.07 billion), and Germany ($734 million) in terms of exports. In comparison, China ($3.42 billion) and Italy ($2.3 billion) were significant importers of animal leather. ^35^ Despite being economically significant for many nations, the tanning industry poses significant environmental challenges owing to solid waste and liquid and gaseous effluents, causing detrimental impacts on air, water, and soil quality.

Solid tannery waste can be divided into removed hair, untreated skin residues, waste from tanned skin, leather trimming, and processed sludge. These residues are abundant and rich in fats ^36^ and proteins ^37^ and depending on their chemical composition, these residues can be recycled for diverse purposes, provided they undergo proper treatment and characterization.

Moreover, regarding water usage in the tanning process, an incredible 50,000 kg of water is required to process just one kilogram of cowhide. ^38^ In addition, tannery effluents are hazardous to decontamination because of their chromium, sulfide, heavy metal, and organic matter content. ^38^^,^^39^ These effluents also exhibit elevated Chemical Oxygen Demand (COD) and Biochemical Oxygen Demand (BOD), ^40^ and even after undergoing advanced chemical and physical treatments, they show low degradability indices. ^41^ In addition, wastewater can permeate through underground layers, as confirmed by research conducted in India, Iran, and Bangladesh, where groundwater samples near tanneries showed high concentrations of Cu, Cr, Pb, Zn, Ni, Al, and As, with Cr registering the highest concentration, ranging from 0.01 to 2.07 mg/L. ^42^^–^^50^ Undoubtedly, this risks ecosystem stability. ^51^

The environmental impact of tannery waste has been under scrutiny for decades, with substantial evidence highlighting the high toxicity of such residues to plants ^52^ and animals ^53^^–^^55^ and bioaccumulation exacerbating this concern. The hazard posed by these residues relies on the presence of contaminating substances, such as sulfides, ^56^ chromium (III), chromium (VI), ^57^^,^^58^ lead, and other heavy metals. The immediate and severe effects of tannery waste on the environment and human health are undeniable. ^59^ In recent years, substantial efforts have been made to remediate, recycle, and reuse various tannery wastes. In this context, repurposing solid tannery waste for glue production is a viable strategy for reducing waste and generating environmentally friendly products within a circular economic framework.

### Historical Perspective of Glue from different natural sources

Adhesives derived from natural sources have had the oldest known historical use in all civilizations for at least 200000 years. ^60^ Evidence of glue residues dates to 1350 BCE, as observed in wood decorations found in King Tutankhamun’s tombs. Additionally, indications of glue usage have been found in ancient civilizations such as Greece and Rome. ^61^ Furthermore, historical records highlight its presence in Edo period paintings in Japan and artifacts from the Joseon dynasty in Korea. ^62^ Today, natural glues have specific services, such as artistic applications, historical conservation, cardboard, packaging ^63^ and the creation of new adhesives.

Historically, glue was primarily produced from animal collagen derived from hides, bones, and connective tissues. Industrially, animal glues come from slaughterhouses that provide animal hides, blood, ^64^^,^^65^ and other sources of proteins ^66^ that can be extracted by hydrolysis. ^67^ Traditionally, to recover animal glue, animal parts, primarily bones from horses, cattle, other livestock, and fishes, are boiled for extended periods in water to obtain collagen, which solidifies into glue upon cooling. ^68^

Other glue sources include water-resistant rennet casein and acidic casein. Rennet casein is produced by coagulating rennets with skim milk at 30°C and acidic casein. ^69^ In contrast, lactic acid casein is derived from inoculating milk with certain bacteria such as *Streptococcus lactis*, *Streptococcus cremoris*, and *Lactococcus lactis* subspecies *cremoris.* ^69^^,^^70^ Chitosan is another natural glue obtained from ground crab and shrimp shell waste, processed through acid or alkali treatment. ^71^^,^^72^ Marine organisms such as mussels, barnacles, and tubeworms secrete protein adhesives that effectively adhere to hydrated underwater surfaces owing to the high proportion of amino acids with phenolic hydroxyl chemical groups. ^73^ These secretions open ample avenues for developing water-resistant adhesives for various purposes.

In Asian cultures, the isinglass is the purest form of fish glue derived from the swim bladder membranes of sturgeons. ^74^ Bone, fish, and hide adhesives have low moisture resistance, which affects the properties of the bond and notably decreases its elasticity and tensile strength. ^75^

Adhesives have been derived from plant resins, saps, natural rubber, ^76^ starches, ^77^ natural gums, latex, soy, lignin, algae, and cellulose. ^78^ Glues from starches such as wheat and rice are commonly used in paper and woodworking applications. The mixture of corn starch with hydrolyzed acrylic emulsion and uzkhitan serves to glue warp threads ^79^ and some starches serve as cohesive elements to create conductive glue for electrode materials. ^80^ The functionality of these glues from starch can be improved with additives; for example, with polymerized lignosulfonates, the water resistance increases. ^81^

**Figure 3.**
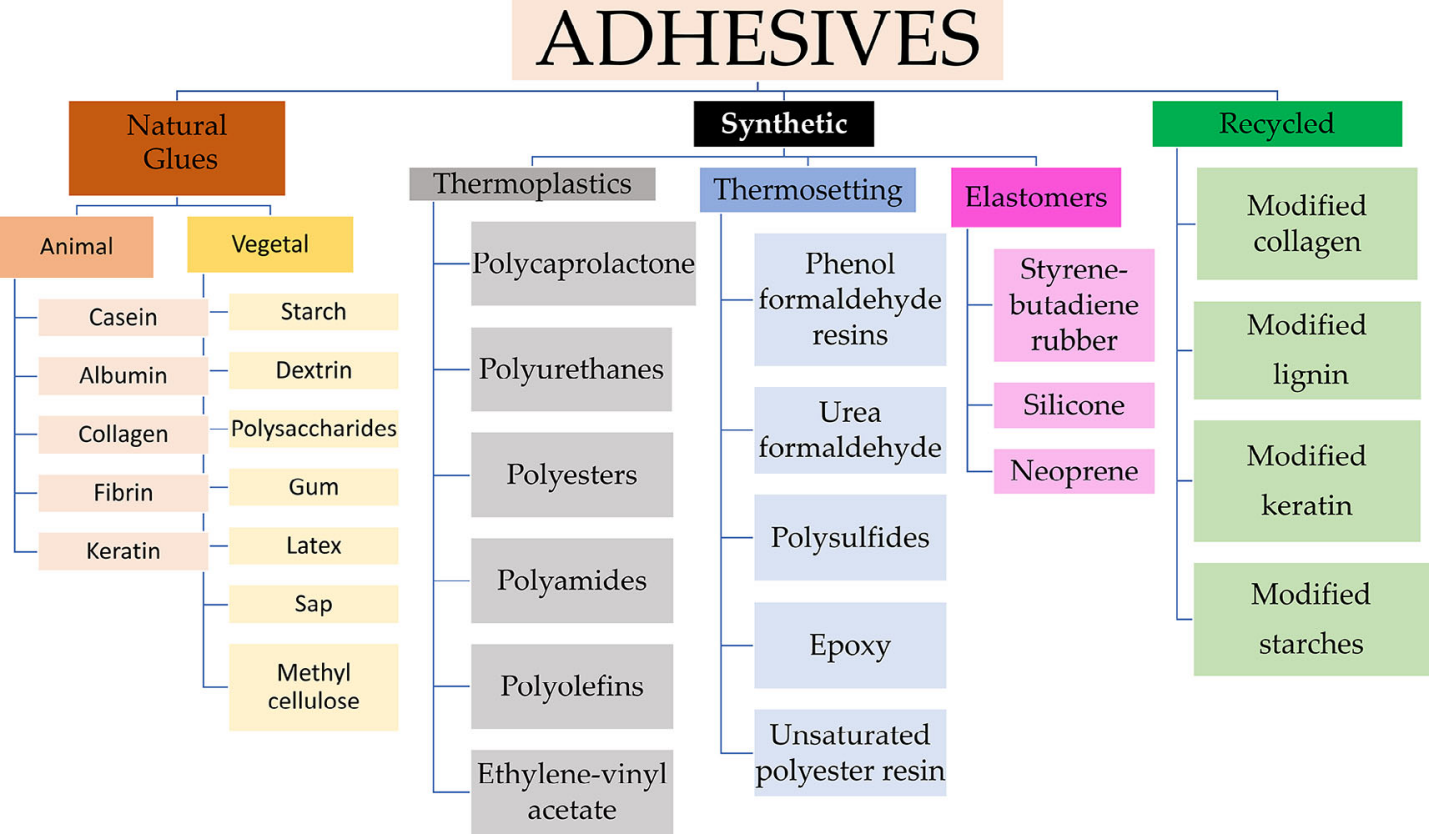
Classification of Adhesives from origin source, highlighting those derived from wastes. ^42^^–^^47^

The adhesive industry has become a specialized field of science, developing numerous innovative adhesive products. To create customized formulas for specific applications, it is crucial to understand the various existing adhesive types. This differentiation forms the basis for the evolution of collagen-modified adhesives. While this review does not delve extensively into the categorization of adhesives, Figure 3 provides a concise overview of the different segments within the adhesive industry, serving as a foundational reference for understanding the different adhesive types.

### Collagen: The protein-based adhesive

With the evolution of industrial processes, there is a need for more efficient and cost-effective adhesives. ^82^ Tanneries, which process animal hides to produce leather, generate significant amounts of waste rich in collagen, particularly trimmings, and shavings, whether tanned or not. ^83^ Rather than discarding these by-products, innovators have realized the potential to utilize this waste for glue production.

Collagen is a complex protein with approximately 28 types. ^84^^,^^85^ This protein is characterized by a unique structure consisting of three parallel polypeptide strands with a left-handed, polyproline II-type (PPII) helical conformation that coils together to form a right-handed triple helix. This structure necessitates that every third residue be glycine, leading to a consistent XaaYaaGly sequence throughout all collagen types. Within this sequence, the amino acids at the Xaa and Yaa positions are (2S)-proline (28%) and (2S,4R)-4-hydroxyproline (38%), respectively, making ProHypGly the predominant triplet, occurring at 10.5% in collagen. ^86^ This formation provides strength and flexibility owing to the high proline and hydroxyproline contents, which prevent the protein from assuming a globular shape. Numerous polar groups in collagen enhance the chain interactions. ^87^^,^^88^

**Figure 4.**
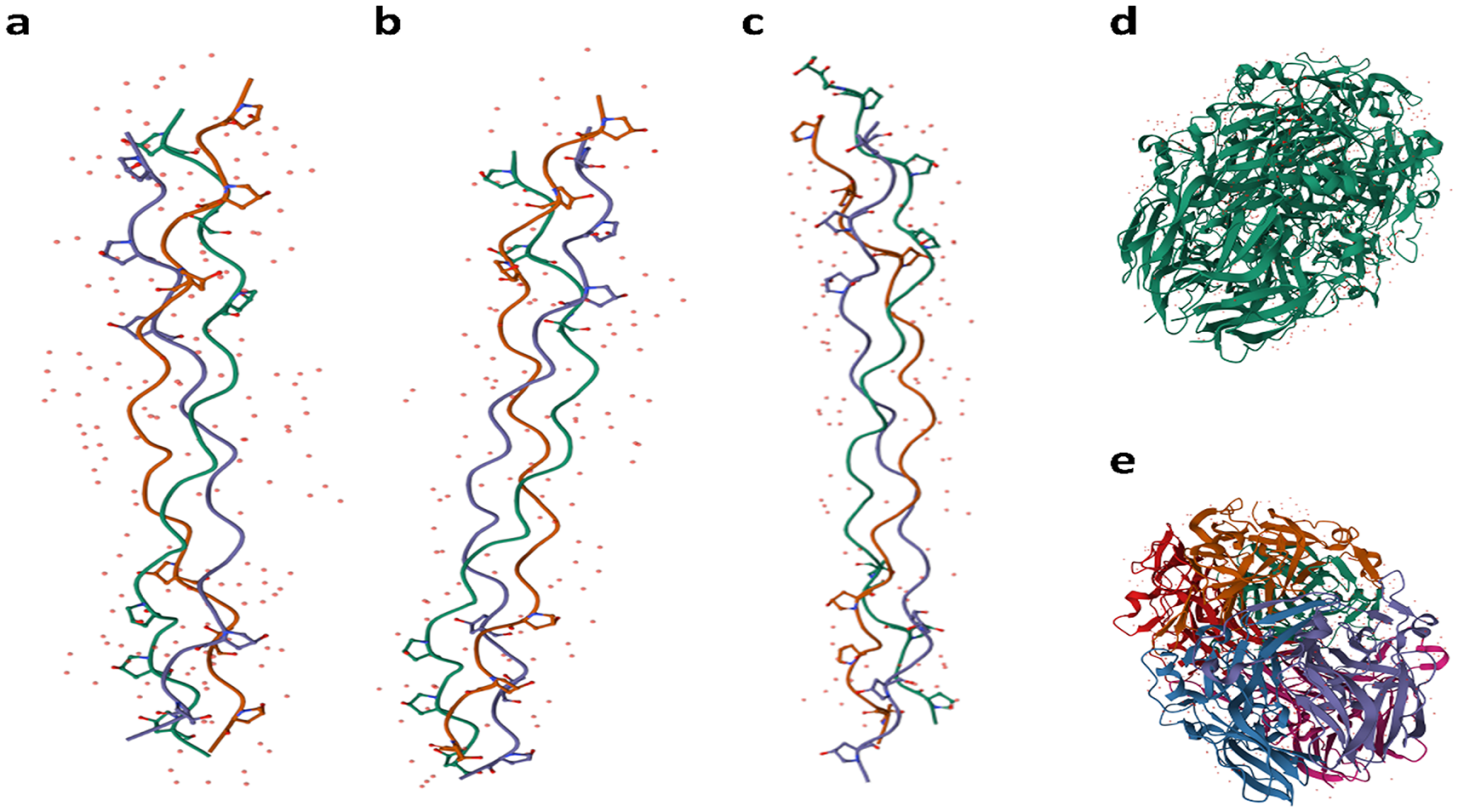
Schematic representation of collagen Types I-V in humans and Bos Taurus. Images are used without modification under the terms of the CC BY 4.0 license—courtesy of PDB-101 ( PDB101.rcsb.org)

The source and preparation method of collagen largely determines its physical, chemical, and mechanical properties. When its chains are shortened, differences originating from various sources diminish, leading to coiled proteins with reduced molecular weight. Native collagen, with a molecular weight of 285–300 KDa, undergoes significant structural changes upon hydrolysis. After denaturation, its triple-helix structure transforms into a random coil form owing to the dissociation of the hydrogen bonds. As a result of this process, hydrolyzed collagen consists of numerous peptides with much lower molecular weights (3–6 KDa). ^89^ The best glues contain collagen Type I because they retain high adherence, ease of gel formation, and an excellent structure to form bonds with other substances compared to simple peptides. Collagen variability is influenced by its origin—whether it comes from skin, connective tissue, cartilage, or bones—as well as by the age and species of the animal, in Figure 4, a schematic representation of five types of collagen is presented. Type I human collagen is predominantly found in the skin, bone, teeth, tendons, ligaments, vascular ligature, and various organs ( Figure 4a). ^90^ Type II collagen is primarily located within cartilage ( Figure 4b), ^91^ while Type III collagen is commonly sourced from the skin, muscles, and blood vessels ( Figure 4c). ^92^ Type IV collagen is present in the epithelial-secreted layer of the basement membrane, as well as the basal lamina ( Figure 4d). ^93^ Type V collagen is a principal component of cell surfaces and the placenta ( Figure 4e). Additionally, Bos Taurus Type IV collagen is depicted in the schematic ( Figure 4e). ^94^

### Collagen-adhesive properties

The adhesion of the protein glue to wood depends on polar and nonpolar group interactions. Amino acids such as glutamic acid, tyrosine, and proline form hydrogen bonds, but these groups often remain inaccessible owing to internal bonds caused by forces such as van der Waals, hydrogen bonds, and hydrophobic interactions. Consequently, basic proteins have limited adhesion and require chemical changes to expose polar protein molecules. ^95^^,^^96^ Furthermore, intramolecular cross-linking is achieved through the oxidative removal of amine groups from specific amino acids within proteins to develop high-strength collagen adhesives. This process leads to the formation of aldehydes, a phenomenon known as the Schiff base protein cross-linking. ^97^

### Hydrolyzed collagen from tanneries

Residues suitable for collagen extraction include untanned skin trimmings and tanned leather shavings. ^98^ Tanneries sell the untanned residues to gelatin factories, which undergo a relatively straightforward transformation.

In contrast, tanned residues, such as wet blue and leather trimmings, require more intricate extraction processes ^99^ because tanned wastes are intertwined collagen strands with agents like tannins, chromium, and alum ^100^ The extraction processes commonly applied to these residues are acid or base hydrolysis at near boiling temperatures or complex enzymatic hydrolysis. ^101^ However, recent investigations have employed multiple combined techniques to enhance collagen yield recovery while mitigating energy consumption ^102^ Collagen extraction typically involves the pre-treatment, hydrolysis, and purification processes.

***Pre-treatment tannery waste***

The main goal of pre-treatment is to disrupt covalent cross-links between collagen molecules because they do not break down even in boiling water. ^103^ Additionally, trimmings and untanned skin must be liberated from chemicals and dirt. These materials are then processed to remove all traces of hair, fat, and flesh, ensuring they can absorb more components for further acid or alkaline treatments. ^104^

Acid Pre-treatment: This method immerses washed and chopped skin pieces in dilute acid. The acid causes the skin to swell and hydrolyze the cross-links. Acid pre-treatment suits fragile skin with less fiber intertwinement, such as porcine and fish skin. ^105^

Alkaline Pre-treatment: Dilute alkalis such as sodium hydroxide, calcium hydroxide, and hydrogen peroxide ^106^ are used. Alkalis is effective for extracting collagen from thick and hard materials. Despite being lengthy, sodium hydroxide treatment is preferred because it swells the skin, aiding alkali diffusion into the tissue matrix. Alkalis also hydrolyses unwanted components. Lower hydroxide concentrations at suitable temperatures retain the acid-soluble collagen and its native structure. ^107^

***Collagen Extraction processes from tannery wastes***

Prolonged boiling for collagen recovery is energy-intensive and inefficient, making it unsuitable for contemporary fabric treatment. Consequently, there is a pressing need to refine the process of extracting collagen from tannery wastes. The most prevalent method for such extraction involves hydrolysis using different agents, as shown in Table 1. There is also a growing interest in advancing novel techniques to enhance extraction yields. Moreover, establishing mathematical-physical models is crucial for improving the efficiency of shaving hydrolysis to reduce the need for extensive experimental trials, as emphasized by Vaskova and Vasek ^108^ whose model introduced a parameter simulation aimed at deriving hydrolyzed collagen from tannery shavings using a flow reactor.

**Table 1.** Summary of collagen extraction processes from tannery wastes and optimal conditions from cited studies.

Methods	Process	Yields, recovery, costs	Advantages	Disadvantages	Cite
Thermal Hydrolysis	Trimmings boil for 3 to 12 hours to dissolve the collagen from hides, bones, and other tissues at 70 °C, at pH 5.5 to 6.0 for 24 hours.	18.25% protein.	Easy control of the process.	Time consuming. High energy consumption. No use of chemicals. Useful for trimmings, not tanned residues.	^109^
The salting-out method	Separate proteins based on their solubility in the presence of salts. NaCl 2.5 M 0.05 M of tris (hydroxymethyl) aminomethane. Suspend precipitate with 0,5 acetic acid and	55 to 65% mass recovery. 1.44 % mass yield	High purity of samples. It is good to obtain collagen type I.	Time consuming. Careful conditioning of extracting baths.	^110^
Isoelectric method	pH adjustment at the correct Isoelectric point, then separation by centrifugation.	55 to 75% mass recovery 2.22 % mass yield	Less purity of samples. Rapid recovery of product.	Keep control of chemical conditions so as not to affect the isoelectric point. Analysis of samples before extraction.	^110^
Alkali method	Water/waste ratio 3:1, 0.5% lime, 85, 8 to 10 hours. Purification with white clay and active carbon.	Nitrogen content 43.84%. 60% recovery.	The most common method. Collagen type I. There are multiple affordable alkalis: MgO, CaO, NaOH, KOH, lime, and ashes.	Collagen extraction needs more purification steps than the enzymatic process.	^111^
Acid method	Water/waste ratio 10:1, Dechroming mix of acids and salting out purification. The process involves stirring a mixture of H _2_C _2_O _4_ (oxalic acid) and H _2_SO _4_ (sulfuric acid) at 250 rpm, 40°C, for 12 hours.	90.6% yield. 95.6% dechroming	High dechroming percentage. Do not destroy the triple helix of Collagen type I. Easy to combine with enzymes.	Medium costs of production. The process needs extra care with pH control.	^112^^–^^114^
Enzymatic method	pH 3.9, 65°C, MgO, alcalase 0.4 % from dry matter. Pepsin immobilized in modified silica clay using 5 5% glutaraldehyde with 5% activated 3-aminopropyltriethoxysilane 25 °C, 90 min, and 3.5 mg ml ^−1^ pepsin.	43% reduction costs	It needs previous extraction via alkalis or acids. Less sludge formation. Fewer purification processes after recovery. We need more investigation to determine ideal conditions for enzymes.	Save costs in energy. Enzymes can diminish the quality of collagen if not extracted from the product. Collagen retains helix structure. Collagen type I.	^115^^,^^116^
Hybrid method Acid-enzyme Alkali enzyme	NaOH/urea solvent system for hydrolysis waste leather shavings (tanned with glutaraldehyde). Cleaning the shavings in a 1% SDS solution, disinfection with 75% sonication. NaOH/urea/H _2_O ratio of 7:12:81 (w/w/w), at a shavings-to-solution percentage of 1:20 (w/w), stirring at 30°C for 6-8 hours. Dialysis 24 hours.	No data	Advanced investigations. For specific uses. Improve removal of chromium. It is the best quality jelly glue.	Dialysis is time-consuming and expensive.	^117^
Ultrasound-enzymatic	Protease from *Bacillus subtilis* with ultrasound.	Conversion ratio 57.6% to 84.1%.	Ultrasound accelerates the enzyme action.	Just for untanned wastes.	^118^
Steam explosion with alkali hydrolysis	CaO for hydrolysis 140°C, 10 min Steam explosion.	30% yield Viscosity at 25°C is 2.4 cP protein solution 24.6 g/L and a molecular mass of 39 kDa.	Steam explosion reduces 36 times the hydrolysis and chromium 96 times	This process liberates low levels of Cr (VI).	^119^

***Post-extraction purification and characterization***

The purification of chromium-extracted collagen is crucial to ensure its suitability for subsequent applications. ^120^ Chromium (III) is the most used as tanning agent. The chrome tanning reaction predominantly targets the carboxyl groups of collagen, which are assumed to be located at the aspartic and glutamic side chains. Kinetics showed quicker Cr (III) reactions with aspartic acid, whereas thermodynamics revealed a stronger tendency of glutamic acid for stable Cr (III) complexes involving Cr-O-Cr bridges. These bridges contribute to bridging the gaps between the collagen chains in the structure of the hide, thereby enabling tanning. ^121^ Hence, the remarkable stability of leather underscores the necessity of devising methods that can disrupt the Cr-O-Cr complex while preserving collagen integrity.

The purification of collagen can be achieved by removing chromium through chemical methods ^122^^,^^123^ such as alkali hydrolysis using 6% magnesium oxide and 1.0% sodium carbonate at 70°C for 48 h, followed by 1% bate enzyme hydrolysis to eliminate almost 80% of chromium from wet blue samples. ^124^ New technologies include ultrasonic dechroming at a maximum of 200 MHz to reduce Cr by 70.2%. ^125^^,^^126^ However, better results were achieved by applying sonication to the waste and adding EDTA at a ratio of 1:3 at 80°C for 30 min to achieve a chromium removal efficiency of 98%. ^127^

These results show that purification of the extracted collagen is possible and practical. The efficiency and yield of dechroming depends on the nature of the waste and the process applied. This field of investigation offers abundant research opportunities and has considerable potential for prolific research.

## Development and comparative analysis of eco-friendly adhesives utilizing collagen recycled from tannery wastes

### Recycled collagen-based adhesives

There are two methods for utilizing natural adhesives. One involves using them directly for their adhesive properties; however, this approach is limited in its applications. Another approach involves blending collagen with other materials to produce copolymers.

Negash et al. fabricated a glue for direct application from a hide-trimming waste. ^128^ through a sequential treatment process involving soaking in a lime solution for four hours, washing, and neutralization with hydrochloric acid. The extraction procedure was conducted under varying conditions from 60 to 70°C for 2.5 to 3.5 hours in a water bath. The optimal conditions were found to be 60°C for three hours. The resulting glue had a viscosity of 90 centipoise, moisture content of 14.6%, ash content of 2.23%, density of 1259 kg/m ^3^, yield of 32%, pH of 5.98, and a shear strength of 260 MN, characteristics that that were superior to the glue used as standard; indicating an optimum extraction process and a standard glue quality for restoration of artistic paintings, sculptures, and other historical artifacts crafted in the past. ^129^ Another advantage is that it is possible to determine the exact composition of these glues. It has become imperative to meticulously analyze the composition of the glues utilized for recreating the original adhesive ^130^ and ensuring meticulous restoration of ancient artworks ^131^^,^^132^ without causing significant alterations that could potentially harm these invaluable pieces.

Formaldehyde-based adhesives have been extensively used owing to their adhesive properties; however, formaldehyde gas emissions present significant health and environmental challenges specially when adopting rigorous emission standards, such as the European Emission Standards E1 and E0. ^133^^,^^134^ One prospective solution combines collagen enzymatically extracted from chromed tannery waste into formaldehyde-based adhesive formulations. Intriguingly, the introduction of hydrolysate at a mass fraction of 0.05 displayed a two-fold increase in methylene bridges compared to methylene-oxide bridges (–CH _2_–O–CH _2_–), as revealed by thermogravimetric analysis. This enhancement implies a potential advantage in mitigating the formation of methylene oxide, a pivotal precursor to formaldehyde emissions, while concurrently enhancing the mechanical attributes of the final product, owing to the augmented molecular mass of the hydrolysate. ^135^ Moreover, the stability of the cross-links observed during condensation in a neutral setting, even when exposed to phthalic acid as a curing agent. ^136^^,^^137^

Sedlicik et al. worked with condensation adhesives based on urea-formaldehyde (UF) mixed with collagen hydrolysate from chrome-tanned leather. The adhesive mixtures were prepared by adding 5% of different modified collagen hydrolysates to the UF adhesive, which underwent condensation at 100°C for a maximum of 45 min. Shear strength testing, EN 314-1, was conducted on beech three-layer plywood after subjecting it to a water-resistance test. The results indicate that all samples meet the required standard values, with a maximum of 2.07 MPa at 19% humidity, although there is a specific decrease in the shear strength compared to the reference sample. Adding collagen hydrolysate within the 3–8% range reduced formaldehyde emissions compared to the commercial reference; this new mixture belongs to the E1 emission class. Experimental evidence, including FT-IR spectroscopy analysis, supports the existence of chemical reactions between the UF resin and collagen hydrolysate ^138^ which is the reason for the improved characteristics of the mix in comparison with hydrolyzed collagen.

Regarding formaldehyde-free adhesives, Islam et al. ^139^ developed a sustainable binding agent for particleboard. This innovative approach yields three distinct adhesive types: collagen-based (Type A), acid extraction (Type B), and PVA/collagen ratio 4:1 (Type C). Notably, adhesive Type C showed a gel time of 4.2 minutes and an impressively high shear strength of 5.31 MPa. Comparatively, adhesive Type B, which lacks additives, exhibited a shear strength of 3.98 MPa. This discrepancy underscores the positive impact of incorporating a modifying agent, indicating that such agents can significantly enhance protein bonding. However, the Type C adhesive performance falls short of the 9.5 MPa exhibited by commercial urea formaldehyde resin. This suggests refining the formulation by adding PVA to increase shear strength.

In another scenario, by fortifying gelatin-based glue with a combination of epoxy-terminated hyperbranched polymers (EHPAE) and sodium dodecyl sulfate, the resulting adhesive showed a remarkable enhancement in shear strength, increasing from 0.92 MPa in pure collagen to 2.285 MPa in the EHPAE-III adhesive. However, it is important to note that this improved shear strength still falls short of the shear strength exhibited by commercially available adhesives (2.469 MPa). The increase in shear strength is attributed to the number of epoxy groups, which establish a robust multipoint cross-linking structure between the molecular chains of gelatin. The EHPAE-III meets the quality requirements of the EN standard for footwear applications. ^140^ Formulation refinement is essential for achieving characteristics equal to or superior to commercial adhesives.

Yang et al. ^141^ conducted a study to create a wood adhesive by combining waterborne polyurethane (WPU) and gelatin (G) derived from residual chromium tannery waste. Through this process, they generated a graft copolymer called WPUG using a method that avoids solvents and involves emulsion copolymerization. They discovered that the adhesive exhibited specific noteworthy characteristics: a considerable dry bonding strength of 4.21 MPa that was recorded at an R-value of 1.5, a contact angle of 111.5°, tensile strength measuring 32.91 MPa, and a temperature exceeding 350°C at which maximum weight loss occurred.

Another approach combines acrylic and hydrolyzed collagen through emulsion copolymerization of acrylic acid and butyl acrylate at various concentrations of hydrolyzed collagen. The resulting acrylic-collagen latex films exhibited changes in their characteristics upon neutralization, becoming sticky and highly flexible, especially the formulation A-C 25 N, containing 25% collagen, demonstrating promising probe track adhesion properties, although the maximum force required to separate the sample, and the stress-strain behavior was 0.0115 MPa. ^142^

When looking for adhesives for a specific application, such as corrugated cardboard, the collagen adhesive CPP-G is produced without water through an anhydrous condensation process driven by mechanical force. The foundation of adhesive CPP-100-2.2 is a collagen-degrading polypeptide-based polymer (CPP) resulting from the synthesis of tricyanogen chloride (TC) and collagen-degrading polypeptides (CDPs) extracted from leather scraps. The research identified that the prime conditions for optimal condensation were a high solubility rate of 97% with an n (TC)/n (CDP) ratio of 2.2 at 100°C. The developed adhesive adhered to the national standard (GB/T6544-2008) S-1.1 grade. The viscosity of CPP-100-2.2 was 0.256 Pa, and its thermal stability ranged from 220°C to 260°C. Notably, the initial adhesion capacity of CPP-G reached 90%, surpassing that of commercial adhesives, and its 48-hour water resistance was comparable to that of a commercial adhesive. ^143^

In these studies, collagen-based adhesives were generally vulnerable to water and tended to redissolve upon heating, posing a drawback for adhesive applications in humid environments. Zhou et al., ^144^ developed a novel adhesive called collagen hydrolysates–silane coupling agent hybrids (CSH). This adhesive uses collagen hydrolysate extracted via the alkali method from leather waste as the base material and three silane coupling agents ((3-glycidyloxypropyl) dimethoxymethylsilane (GPDMS), (3-glycidyloxypropyl) trimethoxysilane (GPTMS), and (3-glycidyloxypropyl) triethoxysilane (GPTES)). The CSH adhesive demonstrated strong adhesive strength and excellent water resistance in tests. Significantly, the water resistance of the adhesive could be increased by modifying the type and quantity of silane coupling agent used. For wood adhesive, the CSH adhesive displayed an impressive dry adhesive strength of 1.57 MPa and a wet adhesive strength of 0.95 MPa, with a maximum cross-linking degree of 43%. ^144^ These results suggest that, given an adequate formulation, collagen hydrolysate derived from tannery waste can potentially serve as a cost-effective and highly efficient adhesive for humid applications.

Biodegradable adhesives can be derived from collagen extracted through alkali hydrolysis combined with magnesium oxide and trypsin from tannery-shaved waste. Extracted collagen was incorporated into biodegradable components, including polyvinyl alcohol and glycerol, at specific concentrations. Using Artificial Neural Network Analysis, the optimal production parameters were determined: working temperature of 65°C, polyvinyl alcohol concentration of 3.2%, and glycerol concentration of 4.2%. The optimal conditions led to an adhesive displaying an experimental peel strength of 12.5 N/mm, higher biodegradability, and similar adhesion power compared to the chemical-based adhesive evaluated in this study. ^145^

Another example of the biodegradability of an adhesive made from collagen is an adhesive consisting of cross-linked collagen and γ-polyglutamic acid. Although this adhesive has a relatively low shear strength of 0.0429 MPa, it displayed a high degree of biodegradation when applied to skin incisions in rats, this adhesive degraded within two weeks in vivo, highlighting the advantages over non-biodegradable petrochemical adhesives. ^146^

A drawback of collagen-based adhesives is their notably low water resistance compared to commercial adhesives. Various studies have explored the incorporation of suitable enhancers to address the water resistance limitations to counteract this issue. For instance, blending collagen with methacrylate, ^147^ incorporating gallic acid into collagen, and ε-polylysine as a bridge, ^148^ melamine-formaldehyde ^149^ or zein ^150^ to mitigate this drawback, but it is recommended more investigation to improve water resistance in collagen-based adhesives.

These studies highlight that collagen can produce diverse adhesives with appropriate additives that will enhance strength, adhesiveness, water resistance, and increased biodegradability.

### Market for collagen and adhesives

The opportunity to manufacture collagen-based adhesives arises at an opportune time, with promising data indicating significant market growth in the hydrolyzed collagen sector, projected to nearly double in value from 2023 to 2032, reaching an estimated $2.34 billion by 2032. ^151^^,^^152^ This is particularly pronounced in North America and Europe, where diverse industries, including food and cosmetics, are heavily invested. Furthermore, the adhesives market ranked as the 281st most traded global commodity, with a trade value of $14.2 billion in 2021. Additionally, the adhesive sector demonstrated an impressive growth rate of 21.3% within a single year, underscoring its dynamic and expanding nature. ^153^^,^^154^ Given the significant growth in the collagen sector, there is potential to establish a sustainable and profitable niche within the global adhesives industry by utilizing tannery waste as a collagen source. This approach could cater to non-edible or medical sectors, although the recovery process costs must be mitigated. The integration could revolutionize the supply chain by aligning waste utilization with market expansion opportunities.

It is feasible and profitable to produce gelatin from chrome shavings in a pilot plant operating with specific equipment, as demonstrated by Cabeza et al. ^155^ In 24 hours, processing 9072 kg of chrome shavings can yield over 900 kg of gelatin daily at approximately $0.52 to $0.57 per kg. Meanwhile, commercially available low-quality gelatins were $3.20 per kg in the same year. ^156^ These studies also illustrated that recovering collagen from tannery wastes can generate additional revenue from the reclaimed chrome and savings related to landfill disposal. In 2023, animal glue prices range between $1.5 to $4 per kilogram, ^157^ making it cost-effective to recycle collagen at an industrial scale even today. ^158^ The competitive price of collagen extracted from tannery wastes makes the industrial production of adhesives and glues possible, especially given the new technology that today is apt to improve collagen yield recovery from tannery wastes, ^159^ as seen in Table 1.

## Utilization and innovations: Tannery waste-derived collagen adhesives applications

In various industries, such as woodwork, textiles, footwear, and packaging, versatile applications of innovative adhesive formulations are making significant strides. These formulations offer promising solutions and advancements for each sector.

As illustrated in Table 2, recycled collagen has been harnessed to produce adhesives suitable for the wood, paper, and textile industries. However, the potential applications of these adhesives extend well beyond the applications. They also hold promise in different arenas where the demand for effective bonding agents is considerable. As technology progresses, these innovative adhesive solutions are anticipated to find novel and unforeseen applications, thereby influencing industries with distinctive attributes and environmentally conscious qualities.

**Table 2.** Patents on Converting Solid Tannery Wastes into Adhesives.

Patent	Description	Application	Cite
CN106753159B Degradable collagen-polyurethane water-based wood adhesive.	Collagen-polyurethane with isocyanate, polyester polyol, hydrophilic chain extender, micromolecular dihydric alcohol chain extender, and neutralizer.	Wood	^160^
CN106800907A A kind of environment-friendly water-based wood adhesive based on degraded collagen solution	Isocyanates and polyester polyol, hydrophilic, glycol chain extenders with degraded collagen from tanneries.	Wood	^161^
CN106753159A One kind of degraded polyurethane aqueous wood adhesive of collagen and preparation method thereof	Chrome shavings with isocyanates and polyester polyol as catalysts.	Wood Paper	^162^
CN109554153A A kind of preparation method and application of collagen base adhesive	Collagen recycled from leather with polyurethane and epoxy resin	Wood and water-resistant applications	^163^
CN110256651A A kind of preparation method of collagen-base paper-making function sizing agent	Hydrolyzed collagen, not necessary from tannery, with diisocyanate, polyalcohol, glycol, hydrophilic chain extender, hydracrylic acid, acid esters, vinyl silicane, persulfate, water-base resin preservative	Paper	^164^
CN111704879A Air-permeable leather adhesive and preparation method thereof	Collagen, polyol, polyisocyanate, chain extender, coupling agent, pore-foaming agent, and a reinforcing agent	Leather	^165^
CN106800907 A kind of environment-friendly water-based wood adhesive based on degraded collagen solution and preparation method thereof	Collagen, isocyanates and polyester polyol, polyurethane prepolymer	Wood	^166^
CN109554153A A kind of preparation method and application of collagen base adhesive	Collagen, polyurethane, epoxy resin	Wood, Paper, Textile	^167^
CN103669109B A kind of preparation method of glue used in paper-making	Hydrolyzed collagen, cross-linking agent	Paper	^168^

## Challenges and Future Directions

There is significant potential for innovation and improvement in collagen adhesives derived from recycled solid waste from tanneries. Exploration of collagen extraction processes, characterization, and modification is necessary to enhance its utility and sustainability. Research efforts should focus on increasing collagen’s adhesion strength, durability, and biodegradability by adding different polymers while considering its compatibility with various substrates, scalability, and commercial viability. ^169^

It is crucial to examine the regulatory compliance and safety of collagen-based adhesives, mainly when used in consumer products like textiles and packaging. By exploring these areas, innovative solutions can be discovered, developing more sustainable, efficient, and versatile collagen adhesives sourced from recycled tannery waste. These efforts can potentially revolutionize industries while aligning them with global sustainability goals.

## Conclusion

This review examines the potential of utilizing collagen extracted from tannery waste for adhesive production and provides a detailed analysis of the extraction methods, formulation techniques, and applications. This study demonstrated the technical feasibility and environmental benefits of this approach.

Research suggests a high level of versatility in using collagen, including blending it with urea-formaldehyde or combining it with waterborne polyurethane for wood-based applications. These blends demonstrated the desired adhesive properties and, in some cases, surpassed those of commercial adhesives. However, collagen-based adhesives are limited by their water resistance. To address this issue, innovations such as the incorporation of silane coupling agents or the addition of other compounds such as methacrylate, gallic acid, ε-polylysine, melamine-formaldehyde, or zein are being explored, indicating a promising future for this field.

However, transitioning from an experimental to a commercial scale remains a challenge. Current investigations are mostly laboratory-level, and comprehensive economic analyses or pilot-scale studies are scarce. Historical data suggests the economic viability of collagen extraction from tannery waste and its subsequent use in adhesive production. However, further consistent and extensive studies are required to confirm this finding.

In essence, environmentally conscious sourcing and the adaptability of collagen are exciting prospects for future adhesive technologies.

## Author contributions

Conceptualization: NEFT; supervision: NEFT and HBM; literature search: NEFT, RDPA and HBM material preparation: NEFT, RDPA and HBM; methodology: NEFT; acquisition of data: NEFT; interpretation of data: NEFT, RDPA; writing—original draft: NEFT, RDPA, HBM; writing—review and final editing: NEFT and HBM; supervision: RDPA, HBM; all authors have read and agreed to the published version of the manuscript.

## Ethical approval

Ethical approval and consent were not required.

## Data Availability

No data are associated with this article.
