# Recycling of collagen from solid tannery waste and prospective utilization as adhesives.

**DOI:** 10.12688/f1000research.155450.2

**Published:** 2025-07-30

**Authors:** Nelly Esther Flores Tapia, Hannibal Brito Moina, Rodny Peñafiel, Lander Vinicio Pérez Aldás

**Affiliations:** 1 Research and Development Directorate, Universidad Técnica de Ambato, Ambato, Tungurahua, Ecuador; 2 Facultad de Ciencias, Escuela Superior Politecnica de Chimborazo, Riobamba, Chimborazo Province, Chimborazo, Ecuador; 3 Food and Biotechnology, Universidad Técnica de Ambato, Ambato, Tungurahua, Ecuador

**Keywords:** collagen; solid tannery wastes; animal glue; collagen adhesives; bio-adhesive, recycled

## Abstract

This study explores the innovative potential of recycled collagen derived from tannery waste for high-performance adhesive formulations. The leather industry generates significant amounts of solid waste, primarily from chromium-tanned leather, which poses substantial environmental challenges. Recent advancements in recycling techniques have opened new avenues for repurposing this waste, particularly through collagen extraction, which comprises about 30-35% of tannery residues. This research systematically reviews the methods and applications of collagen extraction, highlighting the material’s versatility and environmental benefits when used as a bio-adhesive. The review identifies key challenges such as low water resistance, shear strength, and adhesiveness in collagen-based adhesives compared to synthetic counterparts. However, innovative solutions are emerging, including incorporating silane coupling agents and cross-linking technologies that significantly improve adhesive water resistance and mechanical properties. Economic analyses further support using tannery waste-derived collagen in adhesive production, aligning with global sustainability goals and reducing reliance on petrochemical-based adhesives. Despite these advancements, transitioning from laboratory research to commercial applications remains a significant challenge. Current studies primarily focus on small-scale experiments, with limited pilot-scale studies available. Nonetheless, the potential for collagen-based adhesives to replace harmful chemicals in industrial applications is promising, especially in sectors requiring biodegradable and non-toxic materials. This review concludes that while significant progress has been made, further research is necessary to overcome existing limitations and fully realize the commercial potential of collagen-based adhesives derived from tannery waste.

## Introduction

**Figure 1.**
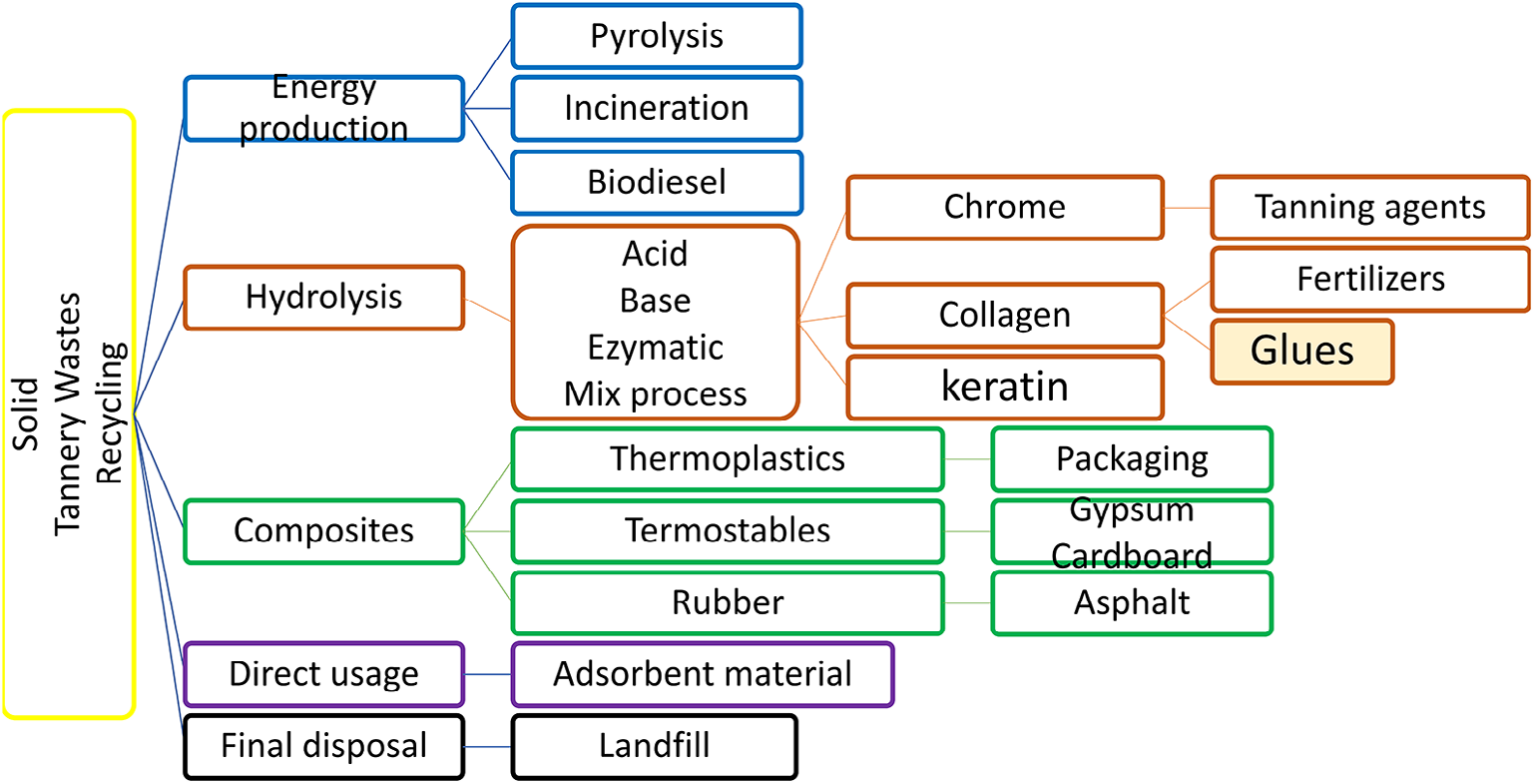
Brief description of processes applied to recycle solid tannery wastes. ^4^^–^^22^

The leather industry, particularly chromium-based tanning, generates substantial solid waste, including chromium sludge, chrome-tanned leather shavings, and trimmings, with only 20% of raw material converted into leather. ^1^^–^^4^ This results in significant collagen-rich waste, which is often discarded in landfills due to the absence of cost-effective recycling programs. ^5^^–^^7^ Solid tannery waste, comprising around 25% untreated skin, contains approximately 30% to 35% collagen and 1.5% chromium, underscoring its potential for resource recovery. ^8^ India alone produces 0.02 million tons of chromium shavings annually (0.8 million tons of chromed leather trimmings per year), indicating a significant potential resource for recycling into valuable products like renewed leather, ^9^ fertilizers in agriculture, composting, ^10^ formulation of composite materials, ^11^^,^^12^ production of biodiesel, ^13^^–^^17^ and extraction of raw materials such as keratin, ^18^ chromium, ^19^ and collagen ^20^^,^^21^ (see Figure 1).

However, synthetic adhesives have largely replaced animal glues due to cost, availability, and consistency concerns. Despite this, synthetic adhesives are highly polluting, non-biodegradable, and dependent on petroleum, underscoring the need for eco-friendly alternatives. Collagen-modified adhesives, especially those derived from tannery waste, present a promising solution by offering both environmental benefits and effective adhesive properties.

Historically, collagen sourced from animal tissues like cartilage and tendons has long been used as an adhesive due to its environmentally friendly and non-toxic properties. ^22^ Over time, animal collagen has been applied in various forms, from craftsmanship to industrial processes, for its natural adhesive qualities. Its composition facilitates efficient and reversible adhesion in applications such as paper and cardboard, with low cure temperatures compared to dispersions or hot melts, setting it apart from synthetic counterparts. ^23^^,^^24^ Despite these advantages, synthetic chemicals supplanted animal glues at the beginning of the 20th century ^25^ due to drawbacks like cost, availability, animal welfare concerns, and inconsistencies in raw material composition that affect adhesive performance. ^26^ While synthetic adhesives provide several benefits, they are highly polluting, non-biodegradable, and reliant on petroleum, driving the search for eco-friendly alternatives. ^27^^–^^29^ Utilizing collagen extracted from tannery waste offers a promising avenue for producing effective adhesives while minimizing waste. ^30^

Although animal glues derived from tannery wastes have been explored as renewable alternatives, their viability is limited by the contamination in tannery waste, rendering them unsuitable for applications like human tissue glues. ^31^^–^^33^ This review evaluates the potential of producing adhesives from collagen extracted from tannery wastes. It explores the methods, applications, and advancements in this area, focusing on extraction techniques, adhesive formulation, and the associated environmental and economic benefits. By addressing gaps in current research, this review provides a comprehensive overview of the challenges and opportunities in utilizing tannery waste for sustainable adhesive production.

## Discussion

### Background on tannery waste and its environmental implications

Global trade in animal leather accounted for only 0.091% of the total international market in 2021, reaching a substantial value of $242.85 billion in 2022. ^34^ Notable exporters in this trade were Italy ($3.55 billion), the United States ($1.88 billion), Brazil ($1.45 billion), China ($1.07 billion), and Germany ($734 million) in terms of exports. In comparison, China ($3.42 billion) and Italy ($2.3 billion) were significant importers of animal leather. ^35^ Despite being economically significant for many nations, the tanning industry poses significant environmental challenges owing to solid waste and liquid and gaseous effluents, causing detrimental impacts on air, water, and soil quality.

Solid tannery waste can be divided into removed hair, untreated skin residues, waste from tanned skin, leather trimming, and processed sludge. These residues are abundant and rich in fats ^36^ and proteins. ^37^ Depending on their chemical composition, they can be recycled for diverse purposes, provided they undergo proper treatment and characterization.

Moreover, regarding water usage in the tanning process, an incredible 50,000 kg of water is required to process just one kilogram of cowhide. ^38^ Also, tannery effluents are hazardous to decontaminating because of their chromium, sulfide, heavy metal, and organic matter content. ^38^^,^^39^ These effluents also exhibit elevated Chemical Oxygen Demand (COD) and Biochemical Oxygen Demand (BOD), ^40^ and even after undergoing advanced chemical and physical treatments, they show low degradability indices. ^41^ In addition, wastewater can permeate through underground layers, as confirmed by research conducted in India, Iran, and Bangladesh, where groundwater samples near tanneries showed high concentrations of Cu, Cr, Pb, Zn, Ni, Al, and As, with Cr registering the highest concentration, ranging from 0.01 to 2.07 mg/L. ^42^^–^^50^ Undoubtedly, this risks ecosystem stability. ^51^

The environmental impact of tannery waste has been under scrutiny for decades, with substantial evidence highlighting the high toxicity of such residues to plants ^52^ and animals ^53^^–^^55^ and bioaccumulation exacerbating this concern. The hazard posed by these residues relies on the presence of contaminating substances, such as sulfides, ^56^ chromium (III), chromium (VI), ^57^^,^^58^ lead, and other heavy metals. The immediate and severe effects of tannery waste on the environment and human health are undeniable. ^59^ In recent years, substantial efforts have been made to remediate, recycle, and reuse various tannery wastes. In this context, repurposing solid tannery waste for glue production is a viable strategy for reducing waste and generating environmentally friendly products within a circular economic framework.

### Historical Perspective of Glue from different natural sources

Adhesives derived from natural sources have had the oldest known historical use in all civilizations for at least 200000 years. ^60^ Evidence of glue residues dates to 1350 BCE, as observed in wood decorations found in King Tutankhamun’s tombs. Additionally, indications of glue usage have been found in ancient civilizations such as Greece and Rome. ^61^ Furthermore, historical records highlight its presence in Edo period paintings in Japan and artifacts from the Joseon dynasty in Korea. ^62^ Today, natural glues have specific services, such as artistic applications, historical conservation, cardboard, packaging, ^63^ and the creation of new adhesives.

Historically, glue was primarily produced from animal collagen derived from hides, bones, and connective tissues. Industrially, animal glues come from slaughterhouses that provide animal hides, blood, ^64^^,^^65^ and other sources of proteins ^66^ that can be extracted by hydrolysis. ^67^ Traditionally, to recover animal glue, animal parts, primarily bones from horses, cattle, other livestock, and fishes, are boiled for extended periods in water to obtain collagen, which solidifies into glue upon cooling. ^68^

Other glue sources include water-resistant rennet casein and acidic casein. Rennet casein is produced by coagulating rennets with skim milk at 30°C and acidic casein. ^69^ In contrast, lactic acid casein is derived from inoculating milk with certain bacteria such as *Streptococcus lactis*, *Streptococcus cremoris*, and *Lactococcus lactis* subspecies *cremoris.* ^69^^,^^70^ Chitosan is another natural glue obtained from ground crab and shrimp shell waste, processed through acid or alkali treatment. ^71^^,^^72^ Marine organisms such as mussels, barnacles, and tubeworms secrete protein adhesives that effectively adhere to hydrated underwater surfaces owing to the high proportion of amino acids with phenolic hydroxyl chemical groups. ^73^ These secretions open ample avenues for developing water-resistant adhesives for various purposes.

In Asian cultures, the isinglass is the purest form of fish glue derived from the swim bladder membranes of sturgeons. ^74^ Bone, fish, and hide adhesives have low moisture resistance, which affects the properties of the bond and notably decreases its elasticity and tensile strength. ^75^

Adhesives have been derived from plant resins, saps, natural rubber, ^76^ starches, ^77^ natural gums, latex, soy, lignin, algae, and cellulose. ^78^ Types of glue from starches such as wheat and rice are commonly used in paper and woodworking applications. The mixture of corn starch with hydrolyzed acrylic emulsion and uzkhitan glue warp threads ^79^ and some starches serve as cohesive elements to create conductive glue for electrode materials. ^80^ The functionality of these glues from starch can be improved with additives; for example, the water resistance increases with polymerized lignosulfonates. ^81^

**Figure 2.**
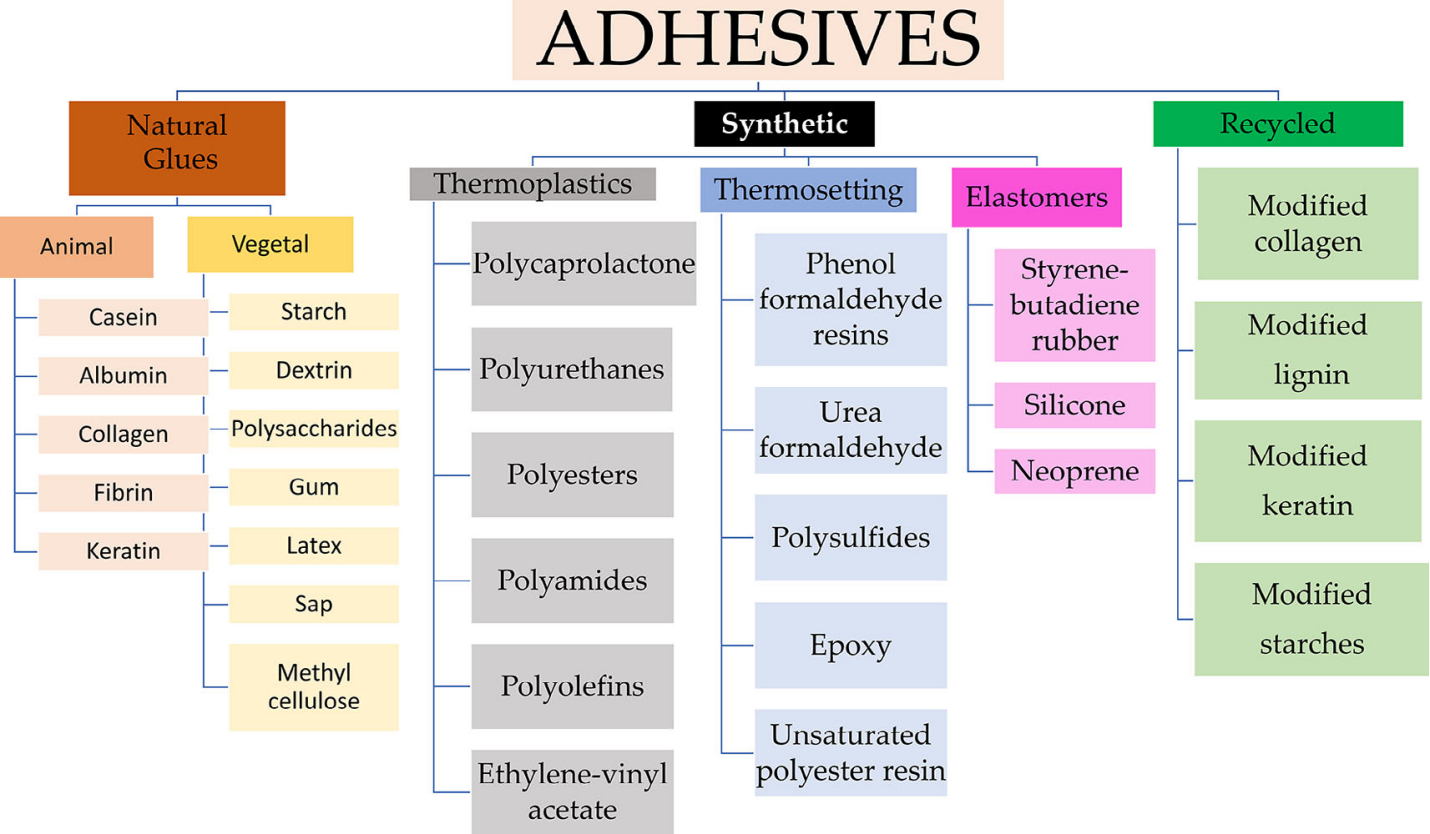
Classification of Adhesives from origin source, highlighting those derived from wastes. ^42^^–^^47^

The adhesive industry has become a specialized field of science, developing numerous innovative adhesive products. To create customized formulas for specific applications, it is crucial to understand the various existing adhesive types. This differentiation forms the basis for the evolution of collagen-modified adhesives. While this review does not delve extensively into the categorization of adhesives, Figure 2 provides a concise overview of the different segments within the adhesive industry, serving as a foundational reference for understanding the different adhesive types.

### Collagen: The protein-based adhesive

With the evolution of industrial processes, there is a need for more efficient and cost-effective adhesives. ^82^ Tanneries, which process animal hides to produce leather, generate significant amounts of waste rich in collagen, particularly trimmings, and shavings, whether tanned or not. ^83^ Rather than discarding these by-products, innovators have realized the potential to utilize this waste for glue production.

Collagen is a complex protein with approximately 28 types. ^84^^,^^85^ This protein is characterized by a unique structure consisting of three parallel polypeptide strands with a left-handed, polyproline II-type (PPII) helical conformation that coils together to form a right-handed triple helix. This structure necessitates that every third residue be glycine, leading to a consistent XaaYaaGly sequence throughout all collagen types. Within this sequence, the amino acids at the Xaa and Yaa positions are (2S)-proline (28%) and (2S,4R)-4-hydroxyproline (38%), respectively, making ProHypGly the predominant triplet, occurring at 10.5% in collagen. ^86^ This formation provides strength and flexibility owing to the high proline and hydroxyproline contents, which prevent the protein from assuming a globular shape. Numerous polar groups in collagen enhance the chain interactions. ^87^^,^^88^

**Figure 3.**
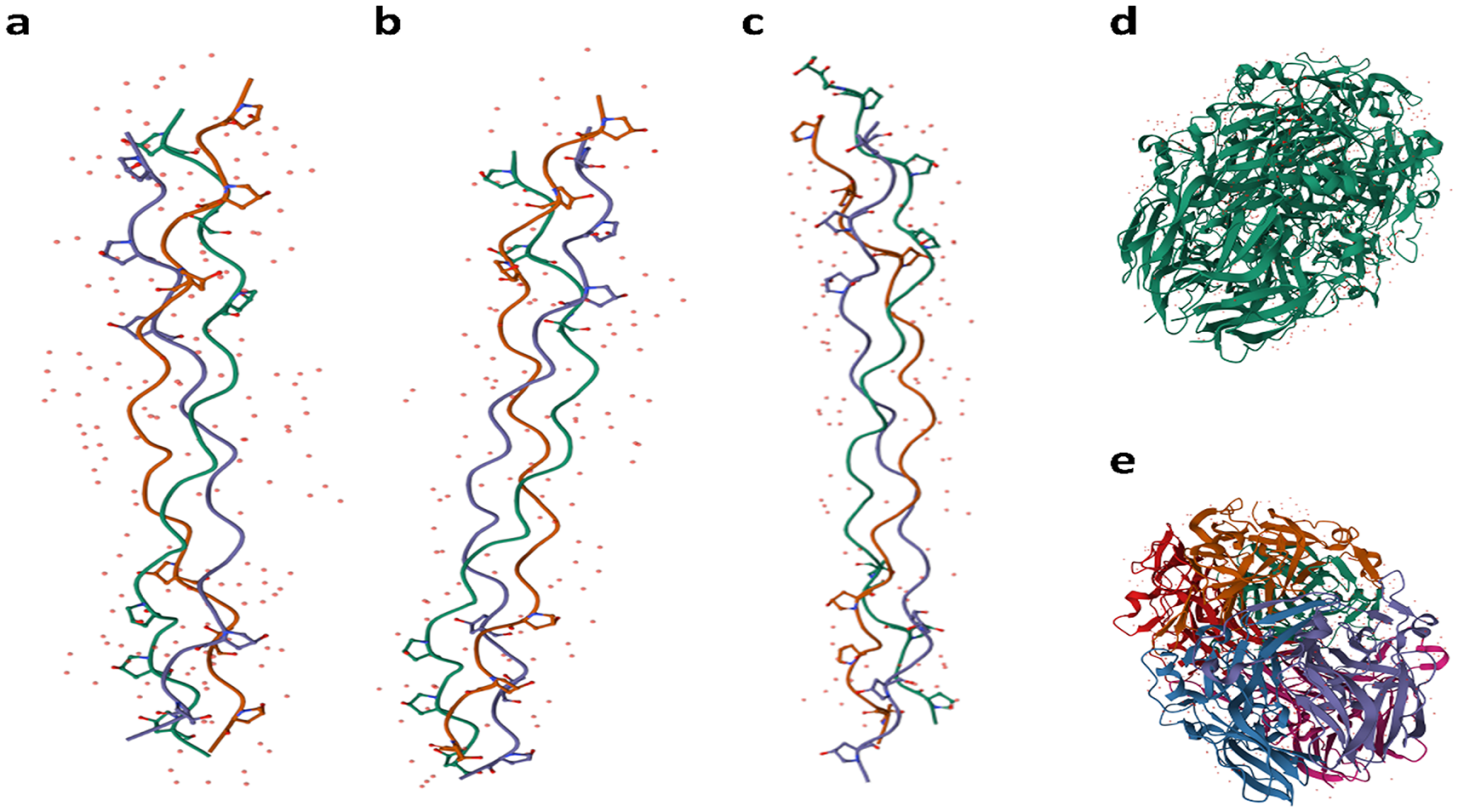
Schematic representation of collagen Types I-V in humans and Bos Taurus. a) Type I human collagen is predominantly found in the skin, bone, teeth, tendons, ligaments, vascular ligature, and various organs ^92^; b) Type II collagen is located within cartilage ^93^; c) Type III collagen is commonly sourced from the skin, muscles, and blood vessels ^94^; d) Type IV collagen is present in the epithelial-secreted layer of the basement membrane as well as the basal lamina ^95^; and e) Type IV Bos Taurus collagen. ^96^ Images are used without modification under the terms of the CC BY 4.0 license—courtesy of PDB-101 ( PDB101.rcsb.org).

The source and preparation method of collagen largely determines its physical, chemical, and mechanical properties. When its chains are shortened, differences originating from various sources diminish, leading to coiled proteins with reduced molecular weight. Native collagen, with a molecular weight of 285–300 KDa, undergoes significant structural changes upon hydrolysis. After denaturation, its triple-helix structure transforms into a random coil form owing to the dissociation of the hydrogen bonds. As a result of this process, hydrolyzed collagen consists of numerous peptides with much lower molecular weights (3–6 KDa). ^89^ The best glues contain collagen Type I because they retain high adherence, ease of gel formation, and an excellent structure to form bonds with other substances compared to simple peptides. The use of collagen is supported by its widespread use in industrial adhesives due to its abundance, strong fibrillar structure, and effective binding properties. ^90^ Proteomic analyses have shown that most animal glues rely mainly on Type I and III collagen extracted from common domestic species, confirming their superior performance over simpler protein fragments. ^91^ Collagen variability is influenced by its origin—whether it comes from skin, connective tissue, cartilage, or bones—as well as by the age and species of the animal, in Figure 3, a schematic representation of five types of collagen is presented. Type I human collagen is predominantly found in the skin, bone, teeth, tendons, ligaments, vascular ligatures, and various organs ( Figure 3a). ^92^ Type II collagen is primarily located within cartilage ( Figure 3b), ^93^ while Type III collagen is commonly sourced from the skin, muscles, and blood vessels ( Figure 3c). ^94^ Type IV collagen is present in the basement membrane’s epithelial-secreted layer and the basal lamina ( Figure 3d). ^95^ Additionally, Bos Taurus Type IV collagen is depicted in the schematic ( Figure 3e). ^96^

### Collagen-adhesive properties

The adhesion of the protein glue to wood depends on polar and nonpolar group interactions. Amino acids such as glutamic acid, tyrosine, and proline form hydrogen bonds. However these groups often remain inaccessible owing to internal bonds caused by forces such as van der Waals, hydrogen bonds, and hydrophobic interactions. Consequently, basic proteins have limited adhesion and require chemical changes to expose polar protein molecules. ^97^^,^^98^ Furthermore, intramolecular cross-linking is achieved through the oxidative removal of amine groups from specific amino acids within proteins to develop high-strength collagen adhesives. This process leads to the formation of aldehydes, a phenomenon known as the Schiff base protein cross-linking. ^99^

### Hydrolyzed collagen from tanneries

Residues suitable for collagen extraction include untanned skin trimmings and tanned leather shavings. ^100^ Tanneries sell untanned residues to gelatin factories, undergoing a relatively straightforward transformation.

In contrast, tanned residues, such as wet blue and leather trimmings, require more intricate extraction processes ^101^ because tanned wastes are intertwined collagen strands with agents like tannins, chromium, and alum. ^102^ The extraction processes commonly applied to these residues are acid or base hydrolysis at near boiling temperatures or complex enzymatic hydrolysis. ^103^ However, recent investigations have employed multiple combined techniques to enhance collagen yield recovery while mitigating energy consumption. ^104^ Collagen extraction typically involves the pre-treatment, hydrolysis, and purification processes.

***Pre-treatment tannery waste***

The main goal of pre-treatment is to disrupt covalent cross-links between collagen molecules because they do not break down even in boiling water. ^105^ Trimmings and untanned skin must be liberated from chemicals and dirt. These materials are then processed to remove all traces of hair, fat, and flesh, ensuring they can absorb more components for further acid or alkaline treatments. ^106^

Acid Pre-treatment: This method immerses washed and chopped skin pieces in dilute acid. The acid causes the skin to swell and hydrolyze the cross-links. Acid pre-treatment suits fragile skin with less fiber intertwinement, such as porcine and fish skin. ^107^

Alkaline Pre-treatment: Dilute alkalis such as sodium hydroxide, calcium hydroxide, and hydrogen peroxide ^108^ are used. Alkalis is effective for extracting collagen from thick and hard materials. Despite being lengthy, sodium hydroxide treatment is preferred because it swells the skin, aiding alkali diffusion into the tissue matrix. Alkalis also hydrolyses unwanted components. Lower hydroxide concentrations at suitable temperatures retain the acid-soluble collagen and its native structure. ^109^

***Collagen Extraction processes from tannery wastes***

Prolonged boiling for collagen recovery is energy-intensive and inefficient, making it unsuitable for contemporary fabric treatment. Consequently, there is a pressing need to refine the process of extracting collagen from tannery wastes. The most prevalent method for such extraction involves hydrolysis using different agents, as shown in Table 1. There is also a growing interest in advancing novel techniques to enhance extraction yields. Moreover, establishing mathematical-physical models is crucial for improving the efficiency of shaving hydrolysis to reduce the need for extensive experimental trials, as emphasized by Vaskova and Vasek ^110^ whose model introduced a parameter simulation aimed at deriving hydrolyzed collagen from tannery shavings using a flow reactor.

**Table 1.** Summary of collagen extraction processes from tannery wastes and optimal conditions from cited studies.

Methods	Process	Yields, recovery, costs	Advantages	Disadvantages	Cite
Thermal Hydrolysis	Trimmings boil for 3 to 12 hours to dissolve the collagen from hides, bones, and other tissues at 70 °C, at pH 5.5 to 6.0 for 24 hours.	18.25% protein.	Easy control of the process.	Time consuming. High energy consumption. No use of chemicals. Useful for trimmings, not tanned residues.	^111^
The salting-out method	Separate proteins based on their solubility in the presence of salts. NaCl 2.5 M 0.05 M of tris (hydroxymethyl) aminomethane. Suspend precipitate with 0,5 acetic acid and	55 to 65% mass recovery. 1.44 % mass yield	High purity of samples. It is good to obtain collagen type I.	Time consuming. Careful conditioning of extracting baths.	^112^
Isoelectric method	pH adjustment at the correct Isoelectric point, then separation by centrifugation.	55 to 75% mass recovery 2.22 % mass yield	Less purity of samples. Rapid recovery of product.	Keep control of chemical conditions so as not to affect the isoelectric point. Analysis of samples before extraction.	^113^
Alkali method	Water/waste ratio 3:1, 0.5% lime, 85, 8 to 10 hours. Purification with white clay and active carbon.	Nitrogen content 43.84%. 60% recovery.	The most common method. Collagen type I. There are multiple affordable alkalis: MgO, CaO, NaOH, KOH, lime, and ashes.	Collagen extraction needs more purification steps than the enzymatic process.	^114^
Acid method	Water/waste ratio 10:1, Dechroming mix of acids and salting out purification. The process involves stirring a mixture of H _2_C _2_O _4_ (oxalic acid) and H _2_SO _4_ (sulfuric acid) at 250 rpm, 40°C, for 12 hours.	90.6% yield. 95.6% dechroming	High dechroming percentage. Do not destroy the triple helix of Collagen type I. Easy to combine with enzymes.	Medium costs of production. The process needs extra care with pH control.	^114^^–^^116^
Enzymatic method	pH 3.9, 65°C, MgO, alcalase 0.4 % from dry matter. Pepsin immobilized in modified silica clay using 5 5% glutaraldehyde with 5% activated 3-aminopropyltriethoxysilane 25 °C, 90 min, and 3.5 mg ml ^−1^ pepsin.	43% reduction costs	It needs previous extraction via alkalis or acids. Less sludge formation. Fewer purification processes after recovery. We need more investigation to determine ideal conditions for enzymes.	Save costs in energy. Enzymes can diminish the quality of collagen if not extracted from the product. Collagen retains helix structure. Collagen type I.	^117^^,^^118^
Hybrid method Acid-enzyme Alkali enzyme	NaOH/urea solvent system for hydrolysis waste leather shavings (tanned with glutaraldehyde). Cleaning the shavings in a 1% SDS solution, disinfection with 75% sonication. NaOH/urea/H _2_O ratio of 7:12:81 (w/w/w), at a shavings-to-solution percentage of 1:20 (w/w), stirring at 30°C for 6-8 hours. Dialysis 24 hours.	No data	Advanced investigations. For specific uses. Improve removal of chromium. It is the best quality jelly glue.	Dialysis is time-consuming and expensive.	^119^
Ultrasound-enzymatic	Protease from *Bacillus subtilis* with ultrasound.	Conversion ratio 57.6% to 84.1%.	Ultrasound accelerates the enzyme action.	Just for untanned wastes.	^120^
Steam explosion with alkali hydrolysis	CaO for hydrolysis 140°C, 10 min Steam explosion.	30% yield Viscosity at 25°C is 2.4 cP protein solution 24.6 g/L and a molecular mass of 39 kDa.	Steam explosion reduces 36 times the hydrolysis and chromium 96 times	This process liberates low levels of Cr (VI).	^121^

***Post-extraction purification and characterization***

The purification of chromium-extracted collagen is crucial to ensure its suitability for subsequent applications. ^122^ Chromium (III) is the most used as a tanning agent. The chrome tanning reaction predominantly targets the carboxyl groups of collagen, which are assumed to be located at the aspartic and glutamic side chains. Kinetics showed quicker Cr (III) reactions with aspartic acid, whereas thermodynamics revealed a stronger tendency of glutamic acid for stable Cr (III) complexes involving Cr-O-Cr bridges. These bridges contribute to bridging the gaps between the collagen chains in the structure of the hide, thereby enabling tanning. ^123^ Hence, the remarkable stability of leather underscores the necessity of devising methods that can disrupt the Cr-O-Cr complex while preserving collagen integrity.

The purification of collagen can be achieved by removing chromium through chemical methods ^124^^,^^125^ such as alkali hydrolysis using 6% magnesium oxide and 1.0% sodium carbonate at 70°C for 48 h, followed by 1% bate enzyme hydrolysis to eliminate almost 80% of chromium from wet blue samples. ^126^ New technologies include ultrasonic dechroming at a maximum of 200 MHz to reduce Cr by 70.2%. ^127^^,^^128^ However, better results were achieved by applying sonication to the waste and adding EDTA at a ratio of 1:3 at 80°C for 30 min to achieve a chromium removal efficiency of 98%. ^129^

These results show that purification of the extracted collagen is possible and practical. The efficiency and yield of dechroming depends on the nature of the waste and the process applied. This field of investigation offers abundant research opportunities and has considerable potential for prolific research.

## Development and comparative analysis of eco-friendly adhesives utilizing collagen recycled from tannery wastes

### Recycled collagen-based adhesives

There are two primary methods for utilizing natural adhesives. One involves their direct use for adhesive purposes, though this approach remains limited in applications. The other combines collagen with additional materials to produce copolymers with enhanced properties. For example, Negash et al. fabricated glue directly from hide-trimming waste ^130^ through a sequential treatment process of lime soaking, washing, and acid neutralization. Extraction between 60 and 70 °C for 2.5–3.5 hours yielded an optimal formulation at 60 °C for three hours. This glue exhibited a viscosity of 90 centipoises, moisture content of 14.6%, ash content of 2.23%, density of 1259 kg/m ^3^, yield of 32%, pH of 5.98, and shear strength of 260 MN—values superior to the reference glue and suitable for restoration of artworks and historical artifacts. ^131^ Moreover, the precise composition of these glues can be determined, which is essential for accurately recreating original adhesives and ensuring careful restoration without compromising valuable pieces. ^132^^–^^134^

Formaldehyde-based adhesives have been widely employed due to their strong bonding performance; however, their use is increasingly restricted because of formaldehyde emissions and stricter regulations such as the European Emission Standards E1 and E0. ^135^^,^^136^ A promising alternative incorporates enzymatically extracted collagen from chromed tannery waste into formaldehyde resin formulations. Introducing hydrolysate at a 5% mass fraction nearly doubled the content of methylene bridges compared to methylene oxide bridges (–CH _2_–O–CH _2_–), as demonstrated by thermogravimetric analysis. ^135^^,^^137^ This change potentially reduces methylene oxide formation—a key precursor of formaldehyde emissions—while enhancing mechanical strength due to increased molecular weight. Cross-link stability was also maintained under neutral conditions and in the presence of phthalic acid as a curing agent. ^138^^,^^139^

Matyašovský et al. ^140^ further demonstrated that collagen hydrolysates modified with urea can substantially reduce formaldehyde emissions when used as additives in urea-formaldehyde (UF) adhesives. By incorporating these collagen components with dialdehyde-modified hardeners, the formaldehyde content in bonded plywood decreased by up to 50% in laboratory tests and approximately 30% in industrial production without significant loss of mechanical strength. These findings reinforce that carefully engineered collagen hydrolysates offer environmental advantages and improve compatibility with UF resins, supporting their use as partial replacements for synthetic adhesives in wood-based applications subject to stringent emission standards.

Building on these insights, Sedlicik et al. ^141^ evaluated UF adhesives modified with collagen hydrolysate from chrome-tanned leather. Adhesives prepared by adding 5% modified hydrolysates underwent condensation at 100 °C for up to 45 minutes. Shear strength testing on beech plywood per EN 314-1 confirmed that all samples met standard requirements, achieving a maximum of 2.07 MPa at 19% humidity, despite a modest reduction compared to unmodified UF resin. Notably, adding 3–8% collagen hydrolysate effectively lowered formaldehyde emissions into the E1 classification, with FT-IR spectroscopy supporting chemical interactions between UF resin and collagen.

To avoid formaldehyde altogether, Islam et al. ^142^ developed sustainable adhesives for particleboard by comparing native collagen (Type A), acid-extracted collagen (Type B), and PVA-crosslinked collagen (Type C). Type C adhesive achieved a gel time of 4.2 minutes and a high shear strength of 5.31 MPa. In contrast, Type B without additives reached 3.98 MPa, demonstrating the benefits of PVA incorporation. However, even this optimized formulation remained below the 9.5 MPa strength typical of commercial urea-formaldehyde resins, suggesting further improvements are needed to match industrial benchmarks.

Additional studies focused on modifying gelatin-based glues with functional polymers, and for example, incorporating epoxy-terminated hyperbranched polymers (EHPAE) and sodium dodecyl sulfate substantially improved shear strength from 0.92 MPa in unmodified gelatin to 2.285 MPa in EHPAE-III adhesives. ^143^ Although performance fell short of the 2.469 MPa achieved by commercial adhesives, this approach fulfilled EN standards for footwear, highlighting the potential of epoxy crosslinking to reinforce protein-based adhesives.

Yang et al. ^144^ produced a wood adhesive by grafting waterborne polyurethane onto gelatin derived from tannery waste. The resulting WPUG copolymer combined high dry bonding strength (4.21 MPa), a contact angle of 111.5°, tensile strength of 32.91 MPa, and excellent thermal stability exceeding 350 °C.

Acrylic–collagen latex adhesives were also developed via emulsion copolymerization with acrylic acid and butyl acrylate, yielding flexible films with tunable properties. Neutralized formulations like A-C 25 N became sticky and highly extensible, demonstrating promising tack adhesion despite relatively low stress–strain performance (0.0115 MPa). ^145^

Liu et al. ^146^ developed a collagen adhesive (CPP-G) through an anhydrous condensation process combining tricyanogen chloride with collagen-degrading polypeptides for corrugated cardboard applications. The optimal product showed a high percentage of solubility rate of 97% and conformed to Chinese standard GB/T6544-2008 S-1.1 grade. Its viscosity (0.256 Pa·s), thermal stability (220–260 °C), and initial adhesion (90%) surpassed many commercial references, while 48-hour water resistance remained comparable.

Despite these advances, many collagen-based adhesives are still vulnerable to water and redissolve upon heating, limiting use in humid conditions. To address this, Zhou et al. ^147^ created collagen hydrolysate–silane coupling agent hybrids (CSH). By combining hydrolysate extracted by alkali hydrolysis with silane crosslinkers (GPDMS, GPTMS, GPTES), they achieved adhesives with dry strength up to 1.57 MPa and wet strength up to 0.95 MPa—exceeding the Chinese Class II plywood standard. These results highlight the importance of crosslinker selection and dosage in improving water resistance.

Biodegradable adhesives incorporating collagen with polyvinyl alcohol and glycerol have also shown promise. Under optimal conditions identified by neural network analysis (65 °C, 3.2% PVA, 4.2% glycerol), peel strength reached 12.5 N/mm, with biodegradability and adhesion comparable to chemical adhesives. ^148^

Finally, Wang et al. ^149^ demonstrated that combining waterborne polyurethane with click-chemistry functional groups significantly improved gelatin adhesives. While plain gelatin had low strength (<0.1 MPa) and poor thermal stability (~310 °C), adding polyurethane increased strength to ~0.17 MPa, and the MWPU-FGE/GE formulation further improved shear strength (~0.68 MPa), peel strength (~1 N/mm), thermal resistance, and wet adhesion. This bio-inspired adhesive outperformed commercial water-based products after extended curing, suggesting a promising sustainable alternative.

Table 2 provides a comprehensive summary of quantitative performance data for a broad range of collagen-based adhesives evaluated in recent years, including their measured bonding strength, viscosity, thermal stability, and water resistance compared to conventional commercial adhesives. The data illustrates that several advanced formulations, particularly those incorporating crosslinkers or chemical modifications, achieve bonding strengths comparable to or exceeding synthetic resin benchmarks in dry conditions. Notably, adhesives based on crosslinked collagen hydrolysates and hybrid systems consistently demonstrate higher mechanical performance than unmodified gelatin or simple protein dispersions.

**Table 2.** Comparative summary of physicochemical and mechanical properties of collagen-derived adhesives versus conventional adhesives.

Type of adhesive	Properties	Commercial adhesive
Recycled collagen-based adhesives ^130^	Direct use from hide-trimming waste. Application: general adhesive applications Viscosity: 90 cP Moisture: 14.6% Ash: 2.23% Density: 1259 kg/m ^3^ Yield: 32% pH: 5.89 Shear strength: (ASTM D2559-04) 260 MN Water Resistance/Solubility: Solubilized completely in hot water (50–55 °C), black color indicates complete solubilization; less soluble in cold water	Direct use from factory. Application: general adhesive applications Viscosity:80 centipoise (cp) Moisture Content: 15.0% Ash Content: 2.0% Density: 1270 kg/m ^3^ Yield: not reported pH: 6.06 Shear Strength: >200 MN Water Resistance: Complete solubility in hot water (50–55 °C), with black color indicating complete dissolution
Collagen in formaldehyde-based adhesives ^137^	DMU + U (fraction 0.05): Endothermic peak 1: ~94 °C, ΔH ≈ 18 J/g Endothermic peak 2: ~125 °C, ΔH ≈ 244 J/g Additional peak at ~60 °C: Not present DMU + U (fraction 0.30): Endothermic peak 1: ~75 °C, ΔH ≈ 30 J/g Endothermic peak 2: ~124 °C, ΔH ≈ 224 J/g Additional peak at ~60 °C: Not present DMU + Hydrolysate (fraction 0.05): Endothermic peak 1: ~92 °C, ΔH ≈ 93 J/g Endothermic peak 2: ~139 °C, ΔH ≈ 174 J/g Additional peak at ~60 °C: Present (associated with bound moisture from the hydrolysate) DMU + Hydrolysate (fraction 0.10): Endothermic peak 1: ~87 °C, ΔH ≈ 105 J/g Endothermic peak 2: ~141 °C, ΔH ≈ 173 J/g Additional peak at ~60 °C: Present DMU + Hydrolysate (fraction 0.50): Endothermic peak 1: ~84 °C, ΔH ≈ 58 J/g Endothermic peak 2: ~137 °C, ΔH ≈ 120 J/g Additional peak at ~60 °C: Present	Pure Urea (U): Endothermic peak 1: ~134 °C, ΔH ≈ 238 J/g Endothermic peak 2: Not present Additional peak at ~60 °C: Not present Pure Dimethylol-Urea (DMU): Endothermic peak 1: ~104 °C, ΔH ≈ 28 J/g Endothermic peak 2: ~126 °C, ΔH ≈ 229 J/g Additional peak at ~60 °C: Not present
Collagen in formaldehyde-based adhesives With phthalic acid ^138^	DMU + 0.05 Hydrolysate + phthalic acid (fraction 0.05): Endothermic peak 1 (TG1): ~25–94 °C, –∆m ≈ 4.1–4.5% Endothermic peak 2 (TG2): ~95–130 °C, –∆m ≈ 4.5–5.4% Endothermic peak 3 (TG3): ~127–151 °C, –∆m ≈ 7.8–10.6% Additional peak at ~150–170 °C (TG4): Present (–∆m ≈ 1.6–5.1%) DMU + 0.05 Hydrolysate + phthalic acid (fraction 0.10): Endothermic peak 1 (TG1): ~25–94 °C, –∆m ≈ 4–5% Endothermic peak 2 (TG2): ~95–130 °C, –∆m ≈ 3.4–4.5% Endothermic peak 3 (TG3): ~127–151 °C, –∆m ≈ 9.8% Additional peak at ~150–170 °C (TG4): Present (–∆m ≈ 3.7–8.3%)	DMU + phthalic acid (fraction 0.01): Endothermic peak 1 (TG1): ~25–94 °C, –∆m ≈ 4–5% Endothermic peak 2 (TG2): ~95–130 °C, –∆m ≈ 5.7% Endothermic peak 3 (TG3): ~127–151 °C, –∆m ≈ 8.2% Additional peak at ~150–170 °C (TG4): Not present DMU + phthalic acid (fraction 0.05): Endothermic peak 1 (TG1): ~25–94 °C, –∆m ≈ 4–5% Endothermic peak 2 (TG2): ~95–130 °C, –∆m ≈ 5.8% Endothermic peak 3 (TG3): ~127–151 °C, –∆m ≈ 8.8% Additional peak at ~150–170 °C (TG4): Present (–∆m ≈ 4.9–5.2%) DMU + phthalic acid (fraction 0.10): Endothermic peak 1 (TG1): ~25–94 °C, –∆m ≈ 4–5% Endothermic peak 2 (TG2): ~95–130 °C, –∆m ≈ 5.7–6.0% Endothermic peak 3 (TG3): ~127–151 °C, –∆m ≈ 8.7% Additional peak at ~150–170 °C (TG4): Present (–∆m ≈ 8.6–9.3%) DMU + 0.05 U + phthalic acid (fraction 0.05): Endothermic peak 1 (TG1): ~25–94 °C, –∆m ≈ 4.2–4.4% Endothermic peak 2 (TG2): ~95–130 °C, –∆m ≈ 5.9% Endothermic peak 3 (TG3): ~127–151 °C, –∆m ≈ 7.9% Additional peak at ~150–170 °C (TG4): Present but low (–∆m ≈ 0.16–0.34%) DMU + 0.05 U + phthalic acid (fraction 0.10): Endothermic peak 1 (TG1): ~25–94 °C, –∆m ≈ 3–4% Endothermic peak 2 (TG2): ~95–130 °C, –∆m ≈ 5.7–7.6% Endothermic peak 3 (TG3): ~127–151 °C, –∆m ≈ 7.8% Additional peak at ~150–170 °C (TG4): Present (–∆m ≈ 3.9–6.5%)
Collagen hydrolysate in urea-formaldehyde adhesives ^138^	UF + 3% Collagen Hydrolysate: pH: 6.2 Curing Time: 74 seconds Dynamic Viscosity (over time): 20 min: 559 mPa·s 590 min: 773 mPa·s Formaldehyde Content: ~3–4 mg/100g (not individually listed but ~30% reduction) Water resistance: > 2MPA UF + 5% Collagen Hydrolysate: pH: 5.8 Curing Time: 77 seconds Dynamic Viscosity: 20 min: 642 mPa·s 590 min: 702 mPa·s Formaldehyde Content: ~3–4 mg/100g Water resistance: > 2MPA UF + 8% Collagen Hydrolysate: pH: 5.5 Curing Time: 76 seconds Dynamic Viscosity: 20 min: 654 mPa·s 590 min: 793 mPa·s Formaldehyde Content: ~3–4 mg/100g Water resistance: > 2MPA	UF Standard: UF resin (Kronores CB 1100) (no collagen) pH: 7.3 Curing Time: 78 seconds Dynamic Viscosity: ~500 mPa·s (very stable over 10 hours) Formaldehyde Content (perforator): 5.2 mg/100g dry board Shear Strength: 2.86 MPa
Melamine-Formaldehyde Adhesive ^140^	VIPOTAR I Modified Adhesive Collagen activator prepared at 20 °C. Optimal dosage: 3.5% of the hardener. Shear strength (Grade 3 conditioning): Average: 2.4 MPa Minimum: 1.7 MPa Maximum: 2.9 MPa Shear strength (Grade 2 conditioning): Average: 2.8 MPa Minimum: 2.4 MPa Maximum: 3.2 MPa Classification: Bond Quality Grade 3, suitable for unlimited outdoor exposure. VIPOTAR II Modified Adhesive Collagen activator prepared at 30 °C. Optimal dosage: 3.5% of the hardener. Shear strength (Grade 3 conditioning): Average: 2.3 MPa Minimum: 1.8 MPa Maximum: 2.8 MPa Shear strength (Grade 2 conditioning): Average: 2.5 MPa Minimum: 2.0 MPa Maximum: 3.0 MPa Classification: Bond Quality Grade 3, suitable for outdoor applications.	KRONOCOL SM 10 (commercial MEF adhesive) Hardener: Conventional commercial hardener (Duslo Šala) Shear strength (Grade 3 conditioning): Average: 1.0 MPa Range: ~0.82–1.26 MPa Classified as Bond Quality Grade 2 (suitable for sheltered exterior conditions).
VIPO, CSIC, and Gelima all are collagen hydrolysates derived from chrome-tanned leather shavings, but they come from different sources and were produced using distinct preparation methods, including enzymatic and chemical hydrolysis. ^141^	Application: modify urea-formaldehyde (UF) and phenol-formaldehyde (PF) resins for plywood bonding Mixture 1a (VIPO hydrolysate + organic acid): Shear Strength: 2.45 MPa Formaldehyde Content (perforator): 2.0 mg/100g dry board Water resistance: > 2MPa Mixture 1b (VIPO hydrolysate + lyotropic + organic acid): Shear Strength: 2.07 MPa Formaldehyde Content: 2.2 mg/100g Water resistance: > 2MPa Mixture 2a (CSIC hydrolysate + organic acid): Shear Strength: 2.53 MPa (highest of all) Formaldehyde Content: 3.7 mg/100g Water resistance: > 2MPa Mixture 2b (CSIC hydrolysate + lyotropic + organic acid): Shear Strength: 2.13 MPa Formaldehyde Content: 3.4 mg/100g Water resistance: > 2MPa Mixture 3a (Gelima hydrolysate + organic acid): Shear Strength: 2.22 MPa Formaldehyde Content: 3.0 mg/100g Water resistance: > 2MPa Mixture 3b (Gelima hydrolysate + lyotropic + organic acid): Shear Strength: 2.07 MPa Formaldehyde Content: 4.3 mg/100g Water resistance: > 2MPa	UF Standard: UF resin (Kronores CB 1100) (no collagen) pH: 7.3 Curing Time: 78 seconds Dynamic Viscosity: ~500 mPa·s (very stable over 10 hours) Formaldehyde Content (perforator): 5.2 mg/100g dry board Shear Strength: 2.86 MPa
Formaldehyde-free collagen adhesives ^142^	Application: adhesives for wood composite panels T-A: Native bone adhesive T-B: Acid-treated bone adhesive (0.5 M H _2_SO _2_) T-C: Acid-treated + PVA crosslinker adhesive T-A (Native bone adhesive) Viscosity: 1.79 Pa·s Moisture: Not specified (adhesive); raw bone ~20.9% Ash: 20.9% in adhesive (after treatment) pH: Not specified Solid Content: 29.8% Gel Time: 16.46 min (very long; unsuitable for production) Glass Transition Temperature (Tg): 57 °C Activation Energy (Ea): 53 kJ/mol Shear Strength: Not tested (failed to bond) Water Resistance: Poor Comparison: Not comparable—did not meet bonding requirements T-B (Acid-treated) Viscosity: 1.26 Pa·s Moisture: Not specified Ash: 13.1% pH: Not specified Solid Content: 41.7% Gel Time: 5.32 min Glass Transition Temperature (Tg): 119 °C Activation Energy (Ea): 74 kJ/mol Shear Strength: ASTM-D905: ~3.68 MPa. EN-205: ~3.4 MPa Water Resistance: WA (24h): 161% TS (24h): 112% T-C (Acid-treated + PVA) Viscosity: 1.06 Pa·s Moisture: Not specified Ash: 14.6% pH: Not specified Solid Content: 43.3% Gel Time: 4.77 min Glass Transition Temperature (Tg): 149 °C Activation Energy (Ea): 78 kJ/mol Shear Strength: ASTM-D905: 5.31 MPa (best among bone adhesives). EN-205: ~5.0 MPa Water Resistance (BTC-2 panel): WA (24h): 143% TS (24h): 93%	UF Resin (Commercial Adhesive, Reference) Viscosity: 0.04 Pa·s (very low) Moisture: Not specified Ash: Not specified pH: 8 Solid Content: 48% Gel Time: 2.30 min (fastest) Glass Transition Temperature (Tg): 152 °C Activation Energy (Ea): 74 kJ/mol Shear Strength:ASTM-D905: 9.43 MPa (highest). EN-205: 8.70 MPa Water Resistance: WA (24h): Lower than bone adhesives (exact % not specified) TS (24h): Lower than bone adhesives Comparison: Best performance in all categories
Gelatin-based glue with EHPAE ^143^	GE Adhesive (unmodified) Solid Content: ~20% Shear Strength: ~1.022 MPa T-Peel Strength: ~1.02 N/mm Water Absorption Rate: ~55% Water Contact Angle: ~54° Comment: Weak adhesion, poor water resistance. GE + SDS Solid Content: ~24% Shear Strength: ~1.5 MPa T-Peel Strength: ~1.8 N/mm Water Absorption Rate: ~50% Water Contact Angle: ~66° Comment: Slight improvement. GE + SDS + EHPAE-I (First Generation Hyperbranched Polymer) Solid Content: ~25% Shear Strength: ~2.29 MPa T-Peel Strength: ~2.54 N/mm Water Absorption Rate: ~40% Water Contact Angle: ~72° Comment: Significant improvement. GE + SDS + EHPAE-II Solid Content: ~27% Shear Strength: ~2.52 MPa T-Peel Strength: ~2.95 N/mm Water Absorption Rate: ~35% Water Contact Angle: ~82° Comment: Better crosslinking. GE + SDS + EHPAE-III (Third Generation Hyperbranched Polymer) Solid Content: ~30% Shear Strength: ~2.65 MPa T-Peel Strength: ~3.38 N/mm Water Absorption Rate: ~26% Water Contact Angle: ~89.6° GE + SDS + Epoxy resin (E-44) Solid Content: ~25% Shear Strength: ~2.10 MPa T-Peel Strength: ~2.60 N/mm Water Absorption Rate: ~40–45% Water Contact Angle: ~65° GE + SDS + PEGDE Solid Content: ~24% Shear Strength: ~2.15 MPa T-Peel Strength: ~2.75 N/mm Water Absorption Rate: ~42% Water Contact Angle: ~70° GE + SDS + Glutaraldehyde Solid Content: ~28% Shear Strength: ~2.80 MPa T-Peel Strength: ~3.00 N/mm Water Absorption Rate: ~32% Water Contact Angle: ~85° GE + SDS + EDC (Carbodiimide) Solid Content: ~27% Shear Strength: ~2.75 MPa T-Peel Strength: ~2.90 N/mm Water Absorption Rate: ~35% Water Contact Angle: ~82°	Loctite E-30CL epoxy resin: Shear Strength: ~3.2 MPa T-Peel Strength: ~2.8 N/mm Water Absorption: Higher than GE/EHPAE adhesives. Dow Corning adhesive: Shear Strength: ~3.0 MPa T-Peel Strength: ~3.1 N/mm Huitian 6302 universal adhesive: Shear Strength: ~2.9 MPa T-Peel Strength: ~2.9 N/mm
Waterborne polyurethane (WPU) and gelatin adhesives ^144^ The adhesives were prepared by grafting gelatin derived from chromium shavings onto waterborne polyurethane, with increasing gelatin content corresponding to R values of 1.5, 3, and 4, resulting in solid contents ranging from ~47% to ~59%. No fillers or additional additives were used	WPUG1.5 (Low gelatin content) Viscosity: 8.43 mPa·s Tensile Strength: 30.52 MPa Elongation at Break: 272% Thermal Stability (TGA): Initial decomposition temperature (Ti): 320.7 °C Peak decomposition temperature (Tp): 384.2 °C Final decomposition temperature (Tf): 412.8 °C Char yield: 17% Water Absorption: 67.68% Water Leaching Rate: 16.57% Gelatin Leaching Rate: 7.38% Contact Angle: 111.5° Dry Bonding Strength: >4.21 MPa Wet Bonding Strength: 1.057 MPa WPUG3 (Medium gelatin content) Viscosity: 12.45 mPa·s Tensile Strength: 32.91 MPa (highest) Elongation at Break: 260.5% Thermal Stability (TGA): Ti: 318.3 °C Tp: 381.9 °C Tf: 410.1 °C Char yield: 21% Water Absorption: 79.76% Water Leaching Rate: 19.96% Gelatin Leaching Rate: 8.31% Contact Angle: ~90° Dry Bonding Strength: 4.16 MPa Wet Bonding Strength: 0.528 MPa WPUG4 (High gelatin content) Viscosity: 24.56 mPa·s Tensile Strength: 14.31 MPa (lowest) Elongation at Break: 83.1% Thermal Stability (TGA): Ti: 310.3 °C Tp: 367.0 °C Tf: 407.6 °C Char yield: 27% Water Absorption: 98.32% Water Leaching Rate: 27.89% Gelatin Leaching Rate: 11.16% Contact Angle: 63° Dry Bonding Strength: 4.09 MPa Wet Bonding Strength: 0.054 MPa	Industrial Gelatin Adhesive (commercial gelatin) Dry Bonding Strength: 3.64 MPa Wet Bonding Strength: Failed completely (adhesive layer detached after soaking) Unmodified Gelatin (G) from chrome shavings Dry Bonding Strength: 1.38 MPa Wet Bonding Strength: Failed completely
Acrylic-collagen adhesives ^145^ Hybrid latexes: A-C 15 (15% collagen) A-C 25 (25% collagen) A-C 35 (35% collagen) A-C 50 (50% collagen) Neutralized versions (A-C 25 N, A-C 50 N)	A-C 15 Collagen Content: 15% Conversion: 67% Particle Diameter: ~734 nm pH: 4.47 Thermal Stability: Tmax: 353 °C T50%: 368 °C Glass Transition: Not reported Mechanical Adhesion (Tack): peak stress 10 MPa and strain at break 1.2 A-C 25 Collagen Content: 25% Conversion: 73% Particle Diameter: ~783 nm pH: 4.52 Thermal Stability: Tmax: 360 °C T50%: 361 °C Glass Transition: Tg1: ~41 °C Mechanical Adhesion (Probe Tack): Mechanical Adhesion (Tack): peak stress 9 MPa and strain at break 2 Saturated film: Tack Force: 4.88 N Tack Area: 0.175 N·mm Dried film: Tack Force: 0.89 N Tack Area: 0.037 N·mm A-C 35 Collagen Content: 35% Conversion: 72% Particle Diameter: ~881 nm pH: 4.24 Thermal Stability: Tmax: 413 °C T50%: 403 °C Glass Transition: Not reported Mechanical Adhesion: Mechanical Adhesion (Tack): peak stress 7 MPa and strain at break 4 A-C 50 Collagen Content: 50% Conversion: 76% Particle Diameter: ~530 nm pH: 4.16 Thermal Stability: Tmax: 399 °C T50%: 398 °C Glass Transition: Tg1: ~42 °C Mechanical Adhesion: Mechanical Adhesion (Tack): peak stress 7 MPa and strain at break 5 A-C 25 N (Neutralized) Based on A-C 25, neutralized with NaOH Glass Transition: Tg1: ~7 °C Tg2: ~27 °C Mechanical Adhesion (Probe Tack): Mechanical Adhesion (Tack): peak stress 1.8 MPa and strain at break 18 Saturated film: Tack Force: 10.05 N (highest among all) Tack Area: 1.970 N·mm Dried film: Tack Force: 0.13 N Tack Area: 0.037 N·mm A-C 50 N (Neutralized) Based on A-C 50, neutralized Glass Transition: Tg1: ~17 °C Tg2: ~24 °C Mechanical Adhesion: Mechanical Adhesion (Tack): peak stress 1.3 MPa and strain at break 10	No lap-shear, peel strength, or tack data for standard adhesives were provided.
Collagen adhesive for corrugated cardboard ^146^ CDP is a collagen degradation product. CPP means Crosslinked Protein Product	CPP-100-2.2 CPP-G Adhesive 50% CPP-100-2.2 5% glucose Small amount of xanthan gum 45% water Moisture content: 35% Viscosity at 50 °C: 9.5 Pa·s Initial adhesion: 90% Bonding strength: 79.6 N/cm ^2^ Water resistance (immersion time): 48 hours GB/T6544-2008 S-1.1 grade “excellent”	Gelatin (Commercial Standard Molecular weight: ~52,000 Viscosity (10% solution): 0.405 Pa·s Thermal stability: similar to CPP-100-2.2 Adhesive (Standard Reference) Moisture content: 35% Viscosity at 50 °C: 10.0 Pa·s Initial adhesion: 95% Bonding strength: 80.3 N/cm ^2^ Water resistance: 48 hours
Collagen hydrolysates–silane coupling agent hybrids ^147^	CH (Collagen Hydrolysate without Crosslinker) Obtained from chrome-tanned leather waste by alkaline hydrolysis. Moisture: 1.42% Ash: 14.73% Chrome oxide content: 0.012% Dry shear strength on birch veneer: 0.91 MPa Wet shear strength: 0.51 MPa Adhesive film observed to be smooth and less adherent after water immersion (poor water resistance). GPDMS–CH Hybrid (crosslinked with (3-glycidyloxypropyl)dimethoxymethylsilane) Crosslinking degree: ~43% Improved surface hydrophobicity (ANS fluorescence index: up to ~91). Dry shear strength: 1.36 MPa Wet shear strength: 0.63 MPa Fracture surface: partially covered by adhesive, somewhat improved water resistance but less than other hybrids. GPTMS–CH Hybrid (crosslinked with (3-glycidyloxypropyl)trimethoxysilane) Crosslinking degree: ~37% Surface hydrophobicity: up to ~366 (significantly higher than GPDMS–CH). Dry shear strength: 1.51 MPa Wet shear strength: 0.95 MPa (exceeds Chinese standard GB/T 9846.3-2004 requirement of 0.7 MPa) Fracture surface: fully covered with adhesive even after soaking, indicating strong adhesion and water resistance. GPTES–CH Hybrid (crosslinked with (3-glycidyloxypropyl)triethoxysilane) Crosslinking degree: ~27% Surface hydrophobicity: up to ~274 Dry shear strength: 1.57 MPa (highest among all samples) Wet shear strength: 0.92 MPa Fracture surface: well covered, high adhesion and water resistance comparable to GPTMS–CH.	Benchmark comparison: These adhesives were compared with Chinese standard GB/T 9846.3-2004 The standard defines shear strength thresholds for adhesives under specified conditions: Minimum Wet Shear Strength Requirement: For interior-use plywood adhesives (Class II): ≥0.7 MPa
Biodegradable collagen adhesives ^148^	Optimized formulation: Working temperature: 65 °C Polyvinyl alcohol concentration: 3.2% Glycerol concentration: 4.2% Peel strength: 12.9 N/mm (measured by SATRA TM 123:1992 method) Shear strength: 4.5 MPa Moisture content: 8–16% (within recommended range) Ash content: 2–5% pH: 5.5–8.0 Viscosity: Not numerically specified but noted to be higher due to added PVA and polyvinyl acetate. Thermal stability: Three-stage degradation observed in TGA, with major protein degradation starting ~220 °C. Water resistance: Not explicitly reported, but peel and shear strength tests suggest good performance.	Standards and references used: Adhesive strength measured using SATRA TM 123:1992 (common in footwear industry). Performance compared to footwear bonding standards (minimum peel strength thresholds: Baby ≥2 N/mm, Child ≥4 N/mm, Women ≥3 N/mm, Men ≥4 N/mm). Benchmark comparison: The authors compared results to prior studies where similar adhesives showed much lower peel strength (~3.25 N/mm).
WPU-GE Adhesive ^149^	WPU-GE Adhesive Solid Content: ~22.05% Shear Strength (RT, 480 min): ~0.1710 MPa T-Peel Strength (RT, 480 min): ~0.2443 N/mm Shear Strength (60 °C, 4 min): Significantly lower than MWPU-FGE/GE Thermal Stability: Onset ~280 °C, main degradation ~320 °C, residue ~25% Main decomposition: ~270–330 °C Morphology: Microcracks and phase separation Comment: Improved over GE but still limited bonding strength MWPU-FGE/GE Adhesive Solid Content: ~28.67% Shear Strength (60 °C, 4 min): ~0.5734 MPa T-Peel Strength (60 °C, 4 min): ~1.0506 N/mm Shear Strength (RT, 480 min): Higher than others T-Peel Strength (RT, 480 min): Highest among the three Thermal Stability: Onset ~300 °C, main degradation ~330 °C, residue ~28% Max decomposition temp: ~300–350 °C Morphology: Dense, smooth fracture surface Comment: Fast curing, best adhesion, excellent water resistance	GE Adhesive (plain gelatin) Solid Content: ~13.77% Shear Strength (RT, 480 min): ~0.0923 MPa T-Peel Strength (RT, 480 min): ~0.1372 N/mm Thermal Stability: Onset ~250 °C, Main degradation ~310 °C, residue ~23% Main decomposition: ~260–300 °C Morphology: Smooth fracture surface, brittle Comment: Weak adhesion, poor water resistance Commercial adhesive: Shear Strength (RT, 480 min): ~0.14 MPa T-Peel Strength (RT, 480 min): ~0.35 N/mm

However, a recurring limitation evident across nearly all compositions is reduced resistance to water exposure. Even adhesives with excellent initial shear or peel strength often show significant loss of cohesion or increased solubility after prolonged immersion or humidity cycling. This variability underscores the importance of further optimizing formulation strategies to enhance water resistance, such as blending hydrophobic resins, introducing moisture-stable crosslinking networks, or applying surface treatments to improve dimensional stability.

These findings are relevant to diverse application areas: certain adhesives are tailored for wood panel bonding, others for corrugated packaging, and some for bio-based composites. Overall, the evidence consolidated here demonstrates that collagen adhesives have significant potential as sustainable alternatives to petrochemical resins. Nevertheless, achieving long-term moisture durability remains the main technical challenge and is critical for future development.

### Market for collagen and adhesives

The opportunity to manufacture collagen-based adhesives arises at an opportune time, with promising data indicating significant market growth in the hydrolyzed collagen sector, projected to nearly double in value from 2023 to 2032, reaching an estimated $2.34 billion by 2032. ^150^^,^^151^ This is particularly pronounced in North America and Europe, where diverse industries, including food and cosmetics, are heavily invested. Furthermore, the adhesives market ranked as the 281st most traded global commodity, with a trade value of $14.2 billion in 2021. Additionally, the adhesive sector demonstrated an impressive growth rate of 21.3% within a single year, underscoring its dynamic and expanding nature. ^152^^,^^153^ Given the significant growth in the collagen sector, there is potential to establish a sustainable and profitable niche within the global adhesives industry by utilizing tannery waste as a collagen source. This approach could cater to non-edible or medical sectors, although the recovery process costs must be mitigated. The integration could revolutionize the supply chain by aligning waste utilization with market expansion opportunities.

It is feasible and profitable to produce gelatin from chrome shavings in a pilot plant operating with specific equipment, as demonstrated by Cabeza et al. ^154^ In 24 hours, processing 9072 kg of chrome shavings can yield over 900 kg of gelatin daily at approximately $0.52 to $0.57 per kg. Meanwhile, the same year, commercially available low-quality gelatins were $3.20 per kg. ^155^ These studies also illustrated that recovering collagen from tannery wastes can generate additional revenue from the reclaimed chrome and savings related to landfill disposal. In 2023, animal glue prices range between $1.5 to $4 per kilogram, ^156^ making it cost-effective to recycle collagen at an industrial scale even today, especially considering the abundance of low-cost leather solid waste, the maturity of hydrolysis-based extraction technologies, and the added value from chromium recovery and landfill cost savings. ^157^ The competitive price of collagen extracted from tannery wastes makes the industrial production of adhesives and glues possible, especially given the new technology that today is apt to improve collagen yield recovery from tannery wastes, ^158^ as seen in Table 1.

## Utilization and innovations: Tannery waste-derived collagen adhesives applications

In various industries, such as woodwork, textiles, footwear, and packaging, versatile applications of innovative adhesive formulations are making significant strides. These formulations offer promising solutions and advancements for each sector.

As illustrated in Table 3, recycled collagen has been harnessed to produce adhesives suitable for the wood, paper, and textile industries. However, the potential applications of these adhesives extend well beyond the applications. They also hold promise in different arenas where the demand for effective bonding agents is considerable. As technology progresses, these innovative adhesive solutions are anticipated to find novel and unforeseen applications, thereby influencing industries with distinctive attributes and environmentally conscious qualities.

**Table 3.** Patents on Converting Solid Tannery Wastes into Adhesives.

Patent	Description	Application	Cite
CN106753159B Degradable collagen-polyurethane water-based wood adhesive.	Collagen-polyurethane with isocyanate, polyester polyol, hydrophilic chain extender, micromolecular dihydric alcohol chain extender, and neutralizer.	Wood	^159^
CN106800907A A kind of environment-friendly water-based wood adhesive based on degraded collagen solution	Isocyanates and polyester polyol, hydrophilic, glycol chain extenders with degraded collagen from tanneries.	Wood	^160^
CN106753159A One kind of degraded polyurethane aqueous wood adhesive of collagen and preparation method thereof	Chrome shavings with isocyanates and polyester polyol as catalysts.	Wood Paper	^161^
CN109554153A A kind of preparation method and application of collagen base adhesive	Collagen recycled from leather with polyurethane and epoxy resin	Wood and water-resistant applications	^162^
CN110256651A A kind of preparation method of collagen-base paper-making function sizing agent	Hydrolyzed collagen, not necessary from tannery, with diisocyanate, polyalcohol, glycol, hydrophilic chain extender, hydracrylic acid, acid esters, vinyl silicane, persulfate, water-base resin preservative	Paper	^163^
CN111704879A Air-permeable leather adhesive and preparation method thereof	Collagen, polyol, polyisocyanate, chain extender, coupling agent, pore-foaming agent, and a reinforcing agent	Leather	^164^
CN106800907 A kind of environment-friendly water-based wood adhesive based on degraded collagen solution and preparation method thereof	Collagen, isocyanates and polyester polyol, polyurethane prepolymer	Wood	^165^
CN109554153A A kind of preparation method and application of collagen base adhesive	Collagen, polyurethane, epoxy resin	Wood, Paper, Textile	^166^
CN103669109B A kind of preparation method of glue used in paper-making	Hydrolyzed collagen, cross-linking agent	Paper	^167^

## Challenges and Future Directions

Gelatin adhesives, despite their environmental benefits and biodegradability, face several limitations that constrain wider industrial use. One primary challenge is their thermal and water stability: gelatin gels are thermoreversible and begin dissolving above ~35–40 °C, limiting applications requiring high thermal resistance or durability in humid conditions such as exterior wood panels or moisture-exposed packaging. ^168^ For this reason, chemical or enzymatic crosslinkers, including glutaraldehyde or transglutaminase, are often incorporated to enhance stability. Mechanically, gelatin-based adhesives exhibit lower strength and dimensional stability than synthetic resins, especially under prolonged stress or humidity. Cost and processing complexity also pose challenges, as high-bloom gelatin entails higher production costs and requires precise control during formulation and application. Regulatory and market acceptance present additional constraints, particularly in food packaging, where animal origin can be a barrier, prompting the development of fish- and plant-derived alternatives that often show lower gel strength. ^169^ Recent studies have proposed adding hydrophobic additives or synthetic polymers to address these deficits to improve moisture resistance, employing enzymatic crosslinking to avoid toxic reagents, and applying bio-catalytic extraction methods to reduce production costs and environmental impact. ^170^ While gelatin offers sustainability advantages, formulation and processing innovation remain essential to make it a competitive alternative to synthetic adhesives.

There is significant potential for improving collagen adhesives derived from recycled tannery waste. Research should focus on optimize extraction, characterization, and modification to enhance adhesion strength, durability, and biodegradability while ensuring compatibility with various substrates, scalability, and commercial viability. ^171^ Regulatory compliance and safety must also be considered, particularly for consumer products like textiles and packaging. Advancing these areas could lead to sustainable, efficient, and versatile adhesives aligned with global sustainability goals.

The main limitations of collagen adhesives—their moisture sensitivity and reduced durability under wet conditions—remain challenges for broader use. These issues stem from the hydrophilicity of collagen’s peptide backbone and its tendency to swell or dissolve in water. Promising strategies to address these issues include chemical crosslinking with glutaraldehyde, epoxides, or silane coupling agents, as shown in Table 2, and improving water resistance by forming covalent networks restricting polymer chain mobility. Blending with hydrophobic polymers such as polyurethanes, polyvinyl acetate, or bio-based resins can further improve dimensional stability and reduce solubility. Surface modification, including water-repellent coatings or nanofillers (e.g., silicas, clays), enhances barrier properties. ^172^^,^^173^ Future work should optimize these strategies to balance mechanical strength, biodegradability, and moisture resistance, making collagen adhesives more competitive for demanding industrial and packaging applications. ^174^

## Conclusion

This review examines the potential of utilizing collagen extracted from tannery waste for adhesive production and provides a detailed analysis of the extraction methods, formulation techniques, and applications. This study demonstrated the technical feasibility and environmental benefits of this approach.

Research suggests a high level of versatility in using collagen, including blending it with urea-formaldehyde or combining it with waterborne polyurethane for wood-based applications. These blends demonstrated the desired adhesive properties and, in some cases, surpassed those of commercial adhesives. However, collagen-based adhesives are limited by their water resistance. To address this issue, innovations such as the incorporation of silane coupling agents or the addition of other compounds such as methacrylate, gallic acid, ε-polylysine, melamine-formaldehyde, or zein are being explored, indicating a promising future for this field.

However, transitioning from an experimental to a commercial scale remains a challenge. Current investigations are mostly laboratory-level, and comprehensive economic analyses or pilot-scale studies are scarce. Historical data suggests the economic viability of collagen extraction from tannery waste and its subsequent use in adhesive production. However, further consistent and extensive studies are required to confirm this finding.

In essence, environmentally conscious sourcing and the adaptability of collagen are exciting prospects for future adhesive technologies.

## Author contributions

Conceptualization: NEFT; supervision: NEFT and HBM; literature search: NEFT, RDPA and HBM material preparation: NEFT, RDPA and HBM; methodology: NEFT; acquisition of data: NEFT; interpretation of data: NEFT, RDPA; writing—original draft: NEFT, RDPA, HBM; writing—review and final editing: NEFT and HBM; supervision: RDPA, HBM; all authors have read and agreed to the published version of the manuscript.

## Ethical approval

Ethical approval and consent were not required.

## Data Availability

No data are associated with this article.
