# Supplementary material for: Recycling of collagen from solid tannery waste and prospective utilization as adhesives
Source: F1000Res. 2025 Jul 30;13:1228. Originally published 2024 Oct 14. [Version 2] doi: 10.12688/f1000research.155450.2 (PMC12906646; doi:10.12688/f1000research.155450.2)
Supplement: Peer review history

# Reviewer report: Reviewer response for version 2

**DOI:** 10.5256/f1000research.179579.r453122

**Published:** 2026-02-14

**Authors:** Majlinda Hylli

**Affiliations:** 1 Polytechnic University of Tirana, Tirana, Albania

The manuscript “Recycling of collagen from solid tannery waste and prospective utilization as adhesives” provides a detailed review of the extraction of collagen from tannery leather waste, its modification and applications, and its relevance to collagen-based adhesives. Instead of focusing on a single methodological or conceptual perspective, the discussion incorporates a wide range of recent studies from materials science, environmental engineering, and industrial chemistry.

 Most factual statements throughout the manuscript are supported by appropriate and credible references, reflecting through engagement with existing literature. The discussion of collagen recovery methods, dechroming strategies, and adhesive formulation, in particular, benefits from well-cited experimental and review studies. 

 The manuscript also demonstrates an alignment with the principles of the circular economy and sustainability goals. The manuscript addresses environmental concerns by exploring the recycling of collagen from solid tannery waste. It highlights the potential use of recovered collagen as an eco-friendly adhesive, which could help to reduce waste and encourage the development of sustainable resources. 

 All the revisions have improved the quality and readability of the manuscript. 

 However, several aspects could be further improved. It would be helpful to indicate whether the references supporting certain numerical data, such as market forecasts, estimated production costs, and the reported performance standards of collagen-based adhesives are recent, authoritative, and directly aligned with the statements made. Are the cited sources up to date and still valid under current market and technological conditions? Do the numerical values remain consistent with the original publications, and have they been verified against the most recent available data? Clarifying these points would significantly strengthen the scientific credibility and reliability of the manuscript.

 Overall, the manuscript is a valuable contribution to the field. It has considerable potential to become a valuable resource for researchers and industry professionals focused on the sustainable valorization of tannery waste.

 So, I approved with minor corrections regarding the questions.

Is the review written in accessible language?

Yes

Are all factual statements correct and adequately supported by citations?

Partly

Are the conclusions drawn appropriate in the context of the current research literature?

Yes

Is the topic of the review discussed comprehensively in the context of the current literature?

Yes

Reviewer Expertise:

Leather

I confirm that I have read this submission and believe that I have an appropriate level of expertise to confirm that it is of an acceptable scientific standard.

# Reviewer report: Reviewer response for version 2

**DOI:** 10.5256/f1000research.179579.r433443

**Published:** 2025-12-24

**Authors:** Marika Gargano

**Affiliations:** 1 University of Naples Federico II, Naples, Italy

The manuscript ***Recycling of collagen from solid tannery waste and prospective utilization as adhesives*** aims to provide a systematic review of the literature concerning the use of recycled collagen derived from solid waste produced by the leather industry as adhesives. The manuscript properly addresses current concerns related to circular economy approaches to waste management and is structured in a comprehensive manner, covering the present status of the leather industry, the use of synthetic and bio-based adhesives, methods for collagen extraction, and its chemical modification for application purposes.

 The study is well organized and aligns with the scope of the journal. The revisions made by the authors have improved the overall quality and readability of the manuscript. Although the abstract and introduction are clear, the Discussion section still requires improvement. Therefore, I recommend acceptance after some revisions, as outlined below:

- The section **“Collagen: The protein-based adhesive”** briefly mentions collagen types but requires further elaboration. The authors should clarify which types of collagen are typically found in which kinds of waste. Moreover, the statement *“The best glues contain collagen Type I…”* needs better contextualization.
- **Table 1** should be revised:
  1. In several rows, advantages and disadvantages are not clearly defined (e.g., in row 6, “Collagen type I” appears under disadvantages, although it is previously described as component of best glues).
  2. The data reported in column 3 should be defined and/or made consistent across entries (indicate which are the differences between “yield”, “mass yield”, “mass recovery”, “recovery” and so on).
- **Table 2** lacks clarity and currently only presents literature data without providing a critical assessment. A more analytical discussion of the reported findings would be beneficial.

Is the review written in accessible language?

Partly

Are all factual statements correct and adequately supported by citations?

Partly

Are the conclusions drawn appropriate in the context of the current research literature?

Yes

Is the topic of the review discussed comprehensively in the context of the current literature?

Yes

Reviewer Expertise:

Leather

I confirm that I have read this submission and believe that I have an appropriate level of expertise to confirm that it is of an acceptable scientific standard, however I have significant reservations, as outlined above.

# Reviewer report: Reviewer response for version 1

**DOI:** 10.5256/f1000research.170636.r359783

**Published:** 2025-01-29

**Authors:** Anke Mondschein

**Affiliations:** 1 FILK Freiberg Institute gGmbH, Freiberg, Saxony, Germany

I fully agree with the issues raised by the first reviewer Ali Yorgancioglu from Ege Üniversitesi, Bornova, Turkey. The topic of the review is very important, but the paper has its scientific limitations. I will not repeat here the topics that have already been addressed and precisely outlined by reviewer 1.

 Additional topics are:

- Literature source missing for the statement that Typ I Col is the best Col type for glueing (p. 7)
- The intended uses of adhesives, technical and medical, should not be mixed and should be clearly separated, ideally with their respective specific challenges for the use of collagen or gelatine as an adhesive. If possible, the market data should also refer to these two very different applications.
- Practical data is lacking, especially for the technical application. Gelatine as an adhesive is already used industrially. It would be instructive to discuss where the actual limits to increased use are (price?, properties?, and how the respective deficits can be overcome)
- Captions should be more precise (e.g. Fig. 4: Explain a - d)

Is the review written in accessible language?

Partly

Are all factual statements correct and adequately supported by citations?

Partly

Are the conclusions drawn appropriate in the context of the current research literature?

Partly

Is the topic of the review discussed comprehensively in the context of the current literature?

Partly

Reviewer Expertise:

Leather

I confirm that I have read this submission and believe that I have an appropriate level of expertise to confirm that it is of an acceptable scientific standard, however I have significant reservations, as outlined above.

# Response

**Published:** 2025-07-01

**Authors:** Nelly Flores

**Affiliations:** DIDE, Universidad Tecnica de Ambato, Ambato, Tungurahua, Ecuador

Reviewer Comment 1:

 Literature source missing for the statement that Type I collagen is the best collagen type for gluing (p.7).

 Response:

 Thank you for highlighting this. We have now added a citation supporting the statement that Type I collagen exhibits superior adhesive properties.

 Reviewer Comment 2:

 The intended uses of adhesives, technical and medical, should not be mixed and should be clearly separated, ideally with their respective specific challenges for the use of collagen or gelatine as an adhesive. If possible, the market data should also refer to these two very different applications.

 Response:

 We agree that distinguishing technical and medical applications improves clarity. In the revised manuscript, we have reorganized the text to create distinct subsections addressing:

 Technical and industrial uses (e.g., wood bonding, paper, packaging)

 Medical applications (e.g., tissue sealants, wound dressings)

 Each subsection now describes the unique performance requirements, regulatory considerations, and market dynamics relevant to that sector. Additionally, references to market data, where available, have been aligned with these categories. We have also removed some examples of medical adhesives that were not directly relevant to recycled collagen, as noted in our prior response.

 Reviewer Comment 3:

 Practical data is lacking, especially for the technical application. Gelatine as an adhesive is already used industrially. It would be instructive to discuss where the actual limits to increased use are (price?, properties?, and how the respective deficits can be overcome).

 Response:

 We appreciate this important observation. In the revised discussion section, we have incorporated a dedicated paragraph elaborating on the limitations of gelatin adhesives in industrial use:

 Their thermoreversible behavior (dissolution above ~35–40 °C)

 Susceptibility to moisture and biodegradation

 Generally lower mechanical strength compared to synthetic resins

 We also discuss price considerations, noting that while gelatin is relatively inexpensive as a byproduct, higher-purity high-bloom gelatin increases costs. Finally, we outline research strategies reported in recent literature for overcoming these deficits, including hydrophobic polymer blending, enzymatic crosslinking, and nanofiller reinforcement (as summarized in Table 2). We believe this provides a more practical perspective on the barriers to wider adoption and possible solutions.

 Reviewer Comment 4:

 Captions should be more precise (e.g., Fig. 4: Explain a–d).

 Response:

 We have revised the caption of Figure 4 to provide explicit descriptions of parts a–d, clarifying the content and purpose of each subfigure. The updated caption now reads:

 Figure 4. Schematic representation of collagen Types I-V in humans and Bos Taurus. a) Type I human collagen is predominantly found in the skin, bone, teeth, tendons, ligaments, vascular ligature, and various organs; b) Type IV collagen is present in the epithelial-secreted layer of the basement membrane as well as the basal lamina

# Reviewer report: Reviewer response for version 1

**DOI:** 10.5256/f1000research.170636.r336497

**Published:** 2024-11-14

**Authors:** Ali Yorgancioglu

**Affiliations:** 1 Ege Üniversitesi, Bornova, Turkey

The manuscript titled as ‘‘ **"Recycling of collagen from solid tannery waste and prospective utilization as adhesives" for F1000Research**” was reviewed.

 I have evaluated the paper in light of the F1000Research scope.  The submission was made as Review Paper. The manuscript discusses the extraction and application of collagen from solid tannery waste as an adhesive. This topic aligns well with current sustainability objectives, particularly within the leather industry. The authors provide a comprehensive review of collagen extraction methods, adhesive formulation, and potential applications, supported by recent advancements in the field.

 However, while the paper is informative, it faces several scientific and structural limitations. Key aspects require refinement to ensure clarity and to meet the publication standards of F1000Research.

 The study addresses an important topic in the circular economy and sustainable waste management. Converting tannery waste into adhesives could reduce landfill waste and minimize reliance on petroleum-based adhesives. This aligns with the increasing need for sustainable materials and methods in various industrial applications.

 While the study is comprehensive in discussing various collagen extraction and adhesive production techniques, it lacks specific experimental data or case studies to support some of its claims. The paper would benefit from quantitative data that compares the performance of collagen-based adhesives with traditional adhesives in specific applications.

 The authors briefly mention the limitations of collagen adhesives, particularly in water resistance and durability. However, more detailed discussion and proposed solutions for overcoming these limitations are necessary to make the paper more practical and applicable to real-world scenarios.

 The manuscript is structured logically, yet certain sections (e.g., Background, Methods) could be more concise. In some parts, especially the discussion on different adhesive applications, the information appears repetitive. A clearer distinction between current findings and future directions could improve readability. Moreover, methods section should be removed. Review papers rarely include a method section since they do not report on original research.

 Author needs to send this manuscript for English proofread as I found some grammatical errors while reviewing the article.

 In the Abstract: "This research systematically reviews the methods and applications of collagen extraction, highlighting the material’s versatility and environmental benefits when used as a bio-adhesive." The phrase “when used as a bio-adhesive” could be simplified to “as a bio-adhesive.”

 In the Methods: "Only documents in English," which should be “Only English-language documents were included.”

 Further editing for grammatical accuracy and flow would enhance the manuscript’s clarity and professionalism.

 After these revision, the paper can be considered publication

Is the review written in accessible language?

Partly

Are all factual statements correct and adequately supported by citations?

Partly

Are the conclusions drawn appropriate in the context of the current research literature?

Partly

Is the topic of the review discussed comprehensively in the context of the current literature?

Partly

Reviewer Expertise:

Leather

I confirm that I have read this submission and believe that I have an appropriate level of expertise to confirm that it is of an acceptable scientific standard, however I have significant reservations, as outlined above.

# Response

**Published:** 2025-07-01

**Authors:** Nelly Flores

**Affiliations:** DIDE, Universidad Tecnica de Ambato, Ambato, Tungurahua, Ecuador

**Reviewer 1**

*Comment:*

 While the study is comprehensive in discussing various collagen extraction and adhesive production techniques, it lacks specific experimental data or case studies to support some of its claims. The paper would benefit from quantitative data that compares the performance of collagen-based adhesives with traditional adhesives in specific applications.

**Response:**

 We fully agree that quantitative comparisons strengthen the discussion. To address this, we incorporated **Table 2**, which provides detailed physicochemical and mechanical properties of a broad range of collagen-derived adhesives and compares them with standard synthetic adhesives and relevant benchmarks. This addition allows readers to see clear performance differences across formulations and applications.

*Comment:*

 The authors briefly mention the limitations of collagen adhesives, particularly in water resistance and durability. However, more detailed discussion and proposed solutions for overcoming these limitations are necessary to make the paper more practical and applicable to real-world scenarios.

**Response:**

 We have expanded the discussion substantially. In the revised manuscript, we now present strategies to overcome water sensitivity and limited durability, including:

- Incorporation of hydrophobic additives and synthetic polymers
- Chemical and enzymatic crosslinking techniques.
- This added section outlines both current approaches and future directions to make collagen adhesives more competitive with conventional resins.

*Comment:*

 The manuscript is structured logically, yet certain sections (e.g., Background, Methods) could be more concise. In some parts, especially the discussion on different adhesive applications, the information appears repetitive. A clearer distinction between current findings and future directions could improve readability. Moreover, methods section should be removed. Review papers rarely include a method section since they do not report on original research.

**Response:**

 We appreciate this suggestion. The *Methods* section has been **removed**, and we restructured the discussion to:

- Consolidate repetitive content about adhesive properties
- Group similar formulations under thematic categories (e.g., urea-formaldehyde, acrylic-collagen)
- Add summary statements distinguishing current evidence and prospective research
  These changes improve conciseness and flow.

*Comment:*

 Author needs to send this manuscript for English proofread as I found some grammatical errors while reviewing the article.

**Response:**

 We have carefully proofread the entire manuscript to improve grammar, consistency, and style. Specific examples highlighted (e.g., “when used as a bio-adhesive”) were corrected as suggested. For example:

- The Abstract was revised to: *"...highlighting the material’s versatility and environmental benefits as a bio-adhesive."*
- The Methods phrase was replaced with: *“Only English-language documents were included.”*
